# The immunopathological landscape of human pre-TCRα deficiency: from rare to common variants

**DOI:** 10.1126/science.adh4059

**Published:** 2024-03-01

**Authors:** Marie Materna, Ottavia M. Delmonte, Marita Bosticardo, Mana Momenilandi, Peyton E. Conrey, Bénédicte Charmeteau-De Muylder, Clotilde Bravetti, Rebecca Bellworthy, Axel Cederholm, Frederik Staels, Christian A. Ganoza, Samuel Darko, Samir Sayed, Corentin Le Floc’h, Masato Ogishi, Darawan Rinchai, Andrea Guenoun, Alexandre Bolze, Taushif Khan, Adrian Gervais, Renate Krüger, Mirjam Völler, Boaz Palterer, Mahnaz Sadeghi-Shabestari, Anne Langlois de Septenville, Chaim A. Schramm, Sanjana Shah, John J. Tello-Cajiao, Francesca Pala, Kayla Amini, Jose S. Campos, Noemia Santana Lima, Daniel Eriksson, Romain Lévy, Yoann Seeleuthner, Soma Jyonouchi, Manar Ata, Fatima Al Ali, Caroline Deswarte, Anaïs Pereira, Jérôme Mégre t, Tom Le Voyer, Paul Bastard, Laureline Berteloot, Michaël Dussiot, Natasha Vladikine, Paula P. Cardenas, Emmanuelle Jouanguy, Mashael Alqahtani, Amal Hasan, Thangavel Alphonse Thanaraj, Jérémie Rosain, Fahd Al Qureshah, Vito Sabato, Marie Alexandra Alyanakian, Marianne Leruez-Ville, Flore Rozenberg, Elie Haddad, Jose R. Regueiro, Maria L. Toribio, Judith R. Kelsen, Mansoor Salehi, Shahram Nasiri, Mehdi Torabizadeh, Hassan Rokni-Zadeh, Majid Changi-Ashtiani, Nasimeh Vatandoost, Hossein Moravej, Seyed Mohammad Akrami, Mohsen Mazloomrezaei, Aurélie Cobat, Isabelle Meyts, Toyofuku Etsushi, Madoka Nishimura, Kunihiko Moriya, Tomoyuki Mizukami, Kohsuke Imai, Laurent Abel, Bernard Malissen, Fahd Al-Mulla, Fowzan Sami Alkuraya, Nima Parvaneh, Horst von Bernuth, Christian Beetz, Frédéric Davi, Daniel C. Douek, Rémi Cheynier, David Langlais, Nils Landegren, Nico Marr, Tomohiro Morio, Mohammad Shahrooei, Rik Schrijvers, Sarah E. Henrickson, Hervé Luche, Luigi D. Notarangelo, Jean-Laurent Casanova, Vivien Béziat

**Affiliations:** 1 Laboratory of Human Genetics of Infectious Diseases, Necker Branch, INSERM, Necker Hospital for Sick Children, Paris, France.; 2 Imagine Institute, University of Paris-Cité, Paris, France.; 3 Laboratory of Clinical Immunology and Microbiology, National Institute of Allergy and Infectious Diseases, National Institutes of Health, Bethesda, USA.; 4 Division of Allergy-Immunology, Department of Pediatrics, Children’s Hospital of Philadelphia; Philadelphia, USA.; 5 University of Paris, Institut Cochin, INSERM U1016, CNRS UMR8104, Paris, France.; 6 Department of Biological Hematology, Hôpital Pitié-Salpêtrière, Assistance Publique-Hôpitaux de Paris (AP-HP) and Sorbonne Université, Paris, France.; 7 Sorbonne University, Paris Cancer Institute CURAMUS, INSERM U1138, Paris, France.; 8 Deptartment of Human Genetics, Dahdaleh Institute of Genomic Medicine, McGill University, Montreal, Quebec, Canada.; 9 Science for Life Laboratory, Department of Medical Biochemistry and Microbiology, Uppsala University, Uppsala, Sweden.; 10 Allergy and Clinical Immunology Research Group, Department of Microbiology, Immunology and Transplantation, KU Leuven, Belgium.; 11 Centogene GmbH, Rostock, Germany.; 12 Human Immunology Section, Vaccine Research Center, National Institute of Allergy and Infectious Diseases, National Institutes of Health, Bethesda, MD, USA.; 13 St. Giles Laboratory of Human Genetics of Infectious Diseases, Rockefeller Branch, The Rockefeller University, New York, USA.; 14 Research Branch, Sidra Medicine, Doha, Qatar.; 15 Helix, San Mateo, CA, USA.; 16 The Jackson Laboratory, Farmington, USA.; 17 Department of Pediatric Respiratory Medicine, Immunology and Critical Care Medicine, Charité - Universitätsmedizin Berlin, corporate member of Freie Universität Berlin, Humboldt-Universität zu Berlin, and Berlin Institute of Health (BIH), Berlin, Germany.; 18 Immunology Research Center, TB and Lung Disease Research Center, Mardaniazar children hospital, Tabriz University of Medical Science, Tabriz, Iran.; 19 Department of Pathology, The Children’s Hospital of Philadelphia, Philadelphia, USA.; 20 Department of Immunology, Genetics and Pathology, Uppsala University and University Hospital, Section of Clinical Genetics, Uppsala, Sweden.; 21 Pediatric Immunology, Hematology and Rheumatology Unit, Necker Hospital for Sick Children, AP-HP, Paris, France.; 22 Cytometry Core Facility, SFR Necker, INSERM US24-CNRS UAR3633, Paris, France.; 23 Department of Pediatric Radiology, University Hospital Necker-Enfants Malades, AP-HP, Paris, France.; 24 Laboratory of Molecular Mechanisms of Hematological Disorders and Therapeutic Implications, INSERM UMR 1163, Paris, France.; 25 Department of Immunology, Complutense University School of Medicine and 12 de Octubre Health Research Institute (imas12), Madrid, Spain.; 26 Department of Translational Genomics, Center for Genomic Medicine, King Faisal Specialist Hospital and Research Center, Riyadh, Saudi Arabia.; 27 Department of Translational Research, Research Division, Dasman Diabetes Institute, Dasman, Kuwait City, Kuwait.; 28 Department of Genetics and Bioinformatics, Research Division, Dasman Diabetes Institute, Dasman, Kuwait City, Kuwait.; 29 Department of Immunology, Allergology and Rheumatology, University of Antwerp, Antwerp University Hospital, Belgium.; 30 Immunology Laboratory, Necker Hospital for Sick Children, Assistance Publique-Hôpitaux de Paris (AP-HP), Paris, France.; 31 Necker Hospital for Sick Children, AP-HP, Paris, France.; 32 Virology, Cochin Hospital, AP-HP, APHP-CUP, Paris, France.; 33 Department of Pediatrics, Department of Microbiology, Immunology and Infectious Diseases, University of Montreal, CHU Sainte-Justine, Montreal, QC, Canada.; 34 Immune System Development and Function Unit, Centro de Biología Molecular Severo Ochoa, Consejo Superior de Investigaciones Científicas (CSIC), Universidad Autónoma de Madrid (UAM), Madrid, Spain.; 35 Division of Gastroenterology, Hepatology and Nutrition at Children's Hospital of Philadelphia.; 36 Cellular, Molecular and Genetics Research Center, Isfahan University of Medical Sciences, Isfahan, Iran.; 37 Department of Genetics and Molecular Biology,Medical School, Isfahan University of Medical Sciences, Isfahan, Iran.; 38 Department of Pediatric Neurology, Children's Medical Center of Abuzar, Jundishapur University of Medical Sciences, Ahvaz, Iran.; 39 Golestan Hospital Clinical Research Development Unit, Ahvaz Jundishapur University of Medical Sciences, Ahvaz, Iran.; 40 Department of Medical Biotechnology, School of Medicine, Zanjan University of Medical Sciences (ZUMS), Zanjan, Iran.; 41 School of Mathematics, Institute for Research in Fundamental Sciences (IPM), Tehran, Iran.; 42 Pediatric Inherited Diseases Research Center, Research Institute for Primordial Prevention of Non-Communicable Disease, Isfahan University of Medical Sciences, Isfahan, Iran.; 43 Neonatal Research Center, Shiraz University of Medical Sciences, Shiraz, Iran.; 44 Medical Genetics Poursina St., Genetic Deptartment, Medical Faculty, Tehran University of Medical Sciences, Tehran, Iran.; 45 Dr. Shahrooei Laboratory, 22 Bahman St., Ashrafi Esfahani Blvd, Tehran, Iran.; 46 Laboratory for Inborn Errors of Immunity, Department of Microbiology, Immunology and Transplantation, Department of Pediatrics, University Hospitals Leuven, KU Leuven, Leuven, Belgium.; 47 Department of Pediatrics, University Hospitals Leuven, KU Leuven, Leuven, Belgium.; 48 Department of Pediatrics and Developmental Biology, Tokyo Medical and Dental University, Tokyo, Japan.; 49 Department of Pediatrics, NHO Kumamoto Medical Center, Kumamoto, Japan.; 50 Department of Pediatrics, National Defense Medical College, Saitama, Japan.; 51 Immunology Center of Marseille-Luminy, Aix Marseille University, Inserm, CNRS, Marseille, France.; 52 Immunophenomics Center (CIPHE), Aix Marseille Université, Inserm, CNRS, Marseille, France.; 53 Department of Anatomy and Cell Biology, College of Medicine, Alfaisal University, Riyadh, Saudi Arabia.; 54 Division of Allergy and Clinical Immunology, Department of Pediatrics, Tehran University of Medical Sciences, Tehran, Iran.; 55 Berlin Institute of Health at Charité – Universitätsmedizin Berlin, Germany.; 56 Berlin-Brandenburg Center for Regenerative Therapies (BCRT), Charité - Universitätsmedizin Berlin, Berlin, Germany.; 57 Labor Berlin GmbH, Department of Immunology, Berlin, Germany.; 58 Center for Molecular Medicine, Department of Medicine (Solna), Karolinska Institute, Stockholm, Sweden.; 59 Department of Human Immunology, Sidra Medicine, Doha, Qatar.; 60 College of Health and Life Sciences, Hamad Bin Khalifa University, Doha, Qatar.; 61 Clinical and Diagnostic Immunology, Department of Microbiology, Immunology, and Transplantation, KU Leuven, Belgium.; 62 Institute for Immunology and Immune Health, University of Pennsylvania; Philadelphia, USA.; 63 Department of Microbiology, Perelman School of Medicine, University of Pennsylvania; Philadelphia, USA.; 64 Department of Pediatrics, Necker Hospital for Sick Children, AP-HP, France.; 65 Howard Hughes Medical Institute, The Rockefeller University, New York, USA.

## Abstract

We describe humans with rare biallelic loss-of-function *PTCRA* variants impairing pre-TCRα expression. Low circulating naïve αβ T cell counts at birth persisted over time, with normal memory αβ and high γδ T cell counts. Their TCRα repertoire was biased, suggesting that noncanonical thymic differentiation pathways can rescue αβ T cell development. Only a minority of these individuals were sick, with infection, lymphoproliferation, and/or autoimmunity. We also report that 1 in 4000 individuals from the Middle East and South Asia are homozygous for a common hypomorphic *PTCRA* variant. They had normal circulating naïve αβ T cell counts but high γδ T cell counts. Although residual pre-TCRα expression drove the differentiation of more αβ T cells, autoimmune conditions were more frequent in these patients than in the general population.

## One-Sentence Summary

Functional αβ T cells and late-onset immunological conditions in humans with rare or common inherited pre-TCRα deficiency.

## Introduction

αβ and γδ T lymphocytes constitute two of the three cellular lineages of adaptive immunity in jawed vertebrates. In a process clarified in mice, they are generated from progenitor stem cells by differentiation in the thymus (1). Double-negative (DN) thymocytes, which lack both CD4 and CD8, are the most immature cells. They differentiate into mature TCRαβ– or TCRγδ–expressing T cells. Cells branch off into these two lineages during early thymopoiesis, which occurs at the same time as *TRD*, *TRG,* and *TRB* locus rearrangements (2-4). Productive *TRD* and *TRG* rearrangements then lead to TCRγδ expression on the cell surface, promoting maturation into γδ T cells. Alternatively, following productive *TRB* locus rearrangement, a TCRβ chain may dimerize with a pre-TCRα protein to generate a pre-TCR. This heterodimer is expressed on the cell surface and, during a process known as β-selection, it promotes a burst of proliferation and differentiation into CD4^+^CD8^+^ double-positive (DP) thymocytes. The *TRA* loci on the DP thymocytes then undergo successive waves of rearrangement (5-8), leading to the expression of TCRαβ heterodimers on the cell surface, and downregulation of the pre-TCRα chain (8, 9). After undergoing negative and positive selection, TCRαβ^+^ thymocytes eventually differentiate into CD4^+^ or CD8^+^ single-positive (SP) mature T cells and migrate to the periphery (10, 11). In four-week-old mice, pre-TCRα loss is associated with a >95% decrease in DP thymocyte counts (12). Although peripheral T cells have not been extensively studied in these mice, only a few TCRαβ cells are detected in lymph nodes (LNs) (5% normal levels), with the cells displaying normal TCR diversity (12, 13). In these studies, the mice remained healthy in pathogen-free conditions but were not challenged with pathogens. They did not develop overt phenotypes but, to our knowledge, no data have been published for *Ptcra*^−/−^ mice beyond the age of 2 months (12-14). The consequences of pre-TCRα deficiency in humans remain unknown. We therefore searched for patients with biallelic germline *PTCRA* variants likely to cause pre-TCRα deficiency.

### Identification of rare biallelic predicted loss-of-function *PTCRA* variants in seven kindreds

**Fig. 1.**
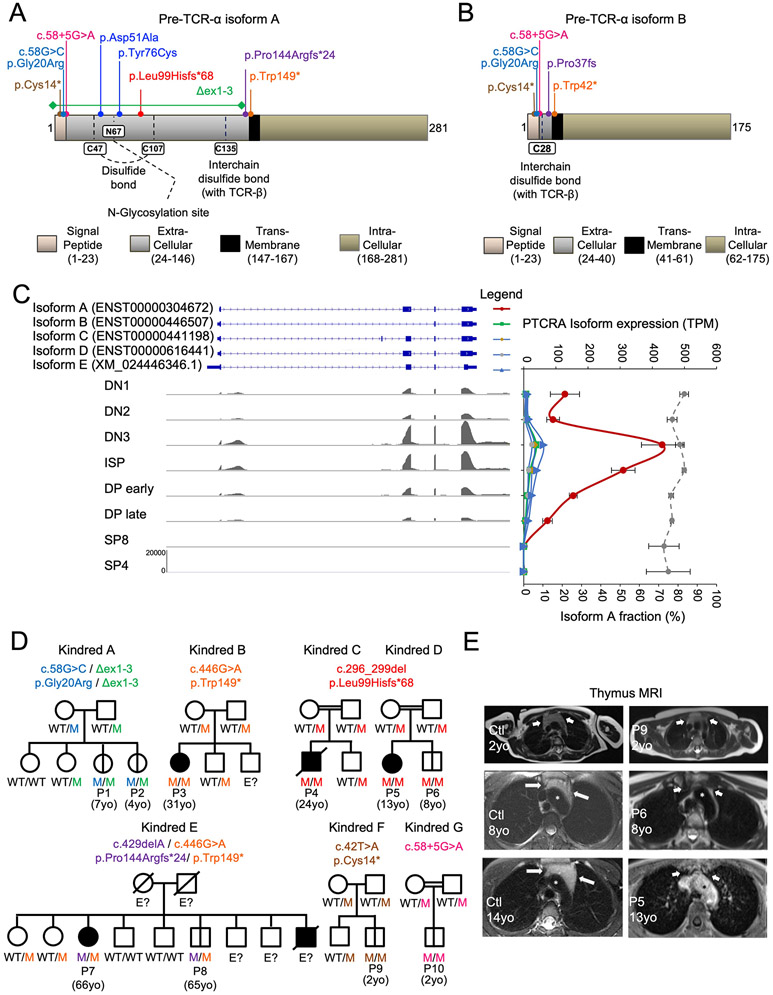
Autosomal recessive pre-TCRα deficiency. (**A** to **B**) Schematic representations of the isoform A (A) and B (B) proteins encoded by *PTCRA*. **(C)** Abundance of the indicated pre-TCRα isoforms in transcripts per million (TPM) across thymocyte developmental stages (DN1, DN2, DN3, ISP, DP early, DP late, and single-positive SP8 and SP4). The proportion of total *PTCRA* transcripts corresponding to isoform A in each thymocyte subset is indicated on the graph (dashed gray line). (**D**) Pedigree of the seven unrelated families displaying familial segregation of the mutant *PTCRA* alleles. The indicated mutant alleles, each with a unique color code, are labeled “M" in the pedigree. Individuals of unknown genotype are labeled “E?”. Asymptomatic individuals are annotated with a vertical bar. (**E**) MRI on axial sections at the level of the aortic arch: T1-weighted sequences after gadolinium injection (P5) and T2-weighted sequences (P6, P9, and controls), for P5, P6, P9, and age- and sex-matched controls. In patients, the thymic lodge, located between the sternum and the aortic arch (asterisk), appears empty (P5 and P6) or small (P9). By contrast, the thymus is clearly visible in controls, even after the onset of puberty (14-year-old girl).

*PTCRA* encodes two functional isoforms in humans and mice (15). Isoform B is 106 amino acids shorter than isoform A and lacks part of the extracellular domain (Fig. 1, A and B). We reanalyzed a public RNAseq dataset corresponding to eight sorted thymocyte subsets from healthy controls (Fig. 1C) (16) and found that isoform A was the principal pre-TCRα isoform in all human thymocyte subsets (Supplementary Text). Unless otherwise specified, we refer below to isoform A. We searched for biallelic predicted loss-of-function (pLOF) variants of the *PTCRA* isoform A including large deletions, frameshift insertions or deletions, premature stop codons, and variants affecting essential splice sites or the start codon. No biallelic pLOF variants meeting these criteria have ever been reported in public databases (17-19). In our in-house database containing data for >25,000 patients, including four other unrelated patients identified by newborn screening (P1, P2, P9, and P10), we identified 10 patients from seven kindreds, all carrying biallelic pLOF variants (Fig. 1D; fig. S1, A to C; and Supplementary Text). The seven pLOF variants in these individuals were present in the homozygous state in five kindreds and in the compound heterozygous state in two kindreds. Five variants were private to the kindreds identified, and two were reported in major public databases, but only in the heterozygous state, with a MAF <10^−4^ (17-19). Two variants were predicted to affect a splice site, two were small frameshift deletions, two led to premature stop codons, and one was a large deletion. The c.58G>C substitution was a missense variant (p.Gly20Arg) but was considered to be pLOF because it was predicted to impair splicing between exons 1 and 2. Apart from the seven pLOF variants identified, only 15 biallelic coding variants, all missense and not predicted to be LOF, were found in public databases or in the HGID in-house database (fig. S1A).

### Clinical features of patients with biallelic pLOF *PTCRA* variants

These 10 patients came from seven unrelated families and were of four different ethnicities (Fig. 1D; data S1; and Supplementary Text). Six of 10 patients with predicted pre-TCRα deficiency, including the four identified by neonatal screening, were clinically asymptomatic at their most recent evaluation (at the ages of 2, 2, 4, 7, 8, and 65 years). The other four patients (13, 24, 31, and 66 years of age) displayed infection, lymphoproliferation, and/or autoimmunity with an onset during their teens or in adulthood (age at onset: 13, 13, 15, and 25 years, respectively). One of these patients died from SARS-CoV-2 pneumonia at the age of 24 years. P9 had a small thymus on MRI at the age of 2 years, whereas P5 and P6 had no visible thymus at the ages of 13 and 8 years, respectively (Fig. 1E). Three of the nine patients with pLOF *PTCRA* variants tested were found to produce autoantibodies, several of which were associated with clinical manifestations (Fig. S2, A to E, and Supplementary Text). Anti-thyroid autoantibodies and/or clinically overt thyroiditis were found in three of the nine patients. P7, who suffered from recurrent herpes infections, had autoantibodies against type I IFNs (Fig. S2A). All known genetic etiologies of these antibodies disrupt T cell tolerance, due to mutations affecting thymocyte development or medullary thymic epithelial cells (20-24).

### Patient alleles cause mRNA decay or premature translational termination

**Fig. 2.**
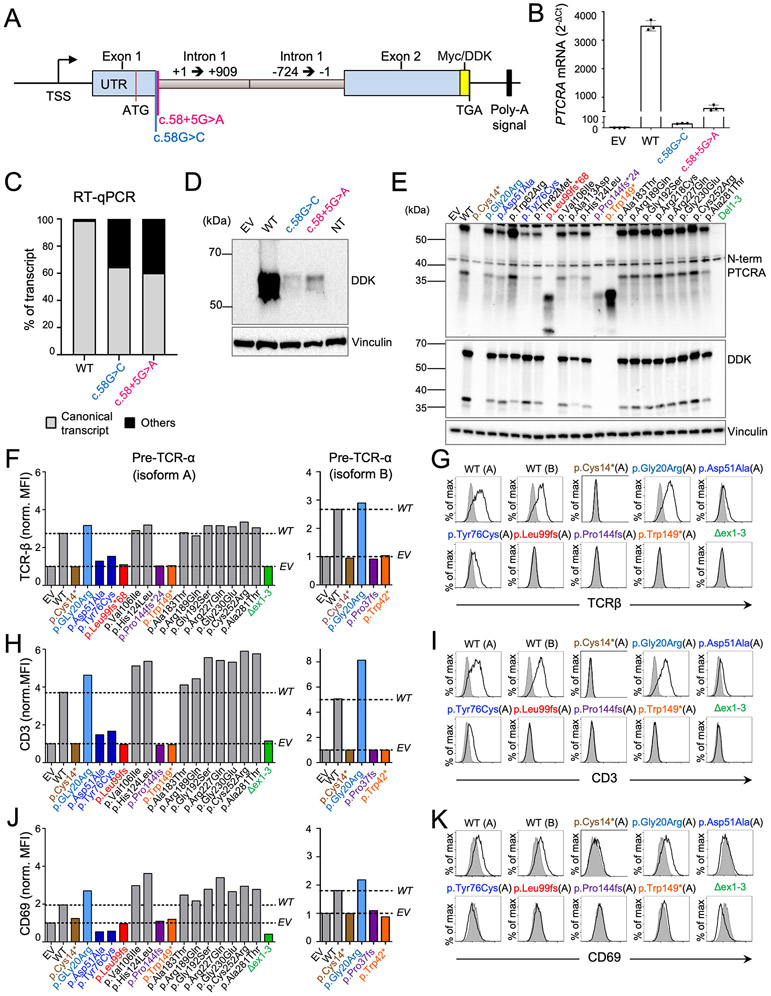
Patient mutations and two mutants from gnomAD are loss-of-function or severely hypomorphic. (**A**) Schematic representation of the artificial gene created to study the splicing between exons 1 and 2 of *PTCRA*. The two mutations tested are depicted. TSS: transcription start site. (**B** to **D**) HEK293T cells were transfected with an empty vector (EV) or with plasmids encoding the artificial gene with the WT or mutant *PTCRA* sequence described in A. (B) The RNA was subjected to RT-qPCR for *PTCRA* with a probe spanning the splice junction between exons 1 and 2. Data are displayed as 2^−ΔCt^ values after normalization relative to an endogenous control (ΔCt). The bar graphs show the mean ± SEM of three technical replicates, and are representative of three independent experiments. (C) Exon trapping. Bar graph showing the proportion of canonical or noncanonical *PTCRA* transcripts in the transfected HEK293T cells. (D) Total protein extracts were subjected to immunoblotting with an antibody against the DDK tag or GAPDH. Data representative of three independent experiments. (**E**) HEK293T cells were transfected with an empty plasmid or with a plasmid carrying a C-terminal DDK-tagged cDNA encoding the WT or the indicated variants of PTCRA isoform A. Total protein extracts were subjected to immunoblotting with an antibody against the DDK tag, pre-TCRα, or Vinculin. Data representative of four independent experiments. (**F** to **K**) TCRα–deficient Jurkat cells were transduced with an EV or with a plasmid encoding the WT isoform A, the WT isoform B or the indicated variant of pre-TCRα. The expression of TCRβ (F and G), CD3ζ (H and I) or CD69 (J and K) at the cell surface was evaluated by flow cytometry on the transduced cell lines. Data representative of three independent experiments. (F, H, and J) Histograms showing the mean fluorescence intensity (MFI) of cells transduced with the indicated *PTCRA* allele normalized against the MFI for EV. (G, I, and K) Representative flow cytometry histogram plot for the indicated *PTCRA* alleles. Cells transduced with the *PTCRA* alleles (black line, unshaded area) are compared to the cells transduced with the EV (shaded).

We then investigated the impact of the pLOF variants on *PTCRA* mRNA and protein. We were unable to test primary cells from the patients because pre-TCRα is expressed only in the thymus. First, using an artificial construct containing the gDNA sequence of *PTCRA* from the 5′ UTR to the end of exon 2 (Fig. 2A), we demonstrated that two of the seven pLOF variants (c.58G>C and c.58+5G>A) severely impaired pre-TCRα expression in vitro by mRNA decay (Fig. 2, B to D; fig. S3A; and Supplementary Text). Second, we transfected HEK293T cells with C-terminally DDK-tagged complementary DNAs (cDNAs) encoding the wild-type (WT) pre-TCRα, one of the six coding pLOF variants identified in the patients, or one of the 15 non-pLOF missense variants identified in the homozygous state in public databases or in our in-house cohort (fig. S1A). Cell extracts were subjected to SDS-PAGE followed by immunoblotting and immunodetection with a monoclonal antibody against DDK- or the N-terminus of pre-TCRα (Fig. 2E; fig. S3B; and Supplementary Text). All variants found in the homozygous state in public databases or in our in-house HGID cohort were normally expressed in this system. By contrast, cDNAs encoding pLOF variants yielded a truncated protein or no protein at all, except for the p.Gly20Arg (c.58G>C) variant, which produced normal amounts of protein in this cDNA overexpression system (Fig. 2E), but was subject to mRNA decay in our artificial gene system (Fig. 2, B to D). Thus, the pLOF variants identified in the patients impair *PTCRA* expression by mRNA decay or premature translation termination.

### Patient variants are loss of function and two variants from public databases are severely hypomorphic

We assessed the ability of the pre-TCRα variants to stabilize TCRβ and CD3 at the cell surface in the TCRα–deficient JR3.11 Jurkat cell line (25, 26). The transduction of these cells with the WT isoform A of pre-TCRα restored the expression of TCRβ and CD3 at the cell surface (Fig. 2, F to I, and fig. S3, C to F). As expected, none of the cDNAs encoding variants from the patients except the cDNA encoding the p.Gly20Arg (c.58G>C) variant restored the cell-surface expression of TCRβ and CD3. By contrast, 13 of the 15 biallelic variants reported in public or in-house databases restored the expression of TCRβ and CD3. The p.Asp51Ala and p.Tyr76Cys variants induced only very low levels of TCRβ and CD3 expression (Fig. 2, F to I, and fig. S3, C to F). Pre-TCR can signal autonomously when expressed at the cell surface. Its successful expression is, therefore, associated with cell-surface expression of the CD69 activation marker (25). Accordingly, all the pre-TCRα-encoding constructs that restored the expression of TCRβ and CD3 at the cell surface also induced weak CD69 expression (Fig. 2, J and K, and fig. S3, G and H). Neither the pLOF variants from the patients nor the p.Asp51Ala and p.Tyr76Cys variants from the public Genome Aggregation Database (gnomAD) V2.1.1 induced CD69 expression on JR3.11 Jurkat cells. Similar findings were obtained when the deleterious variants were tested for their impact on isoform B (Fig. 2, F to K, and fig. S3, C, D, and G). Thus, the seven alleles from the patients are biochemically LOF and the patients are predicted to have an autosomal recessive, complete form of pre-TCRα deficiency. Moreover, two missense variants (p.Asp51Ala and p.Tyr76Cys) found in the homozygous state in the general population are highly deleterious for pre-TCRα function.

### Population genetics of the Asp51Ala and Tyr76Cys variants

The p.Asp51Ala and p.Tyr76Cys variants identified in gnomAD affect residues interacting with TCRβ (fig. S4A) (27). The p.Asp51Ala variant affects a charged residue in the extracellular domain. In the mouse, knock-in mutations of such residues impair the interaction between pre-TCRα and TCRβ, leading to a decrease in the count of DP thymocytes and an increase in γδ T cell counts (28, 29). The p.Asp51Ala and p.Tyr76Cys variants may, therefore, impair dimerization between pre-TCRα and TCRβ. In gnomAD v2.1.1 and the Centogene Biodatabank, the pTyr76Cys variant was most frequent in sub-Saharan Africans, with a MAF of ~0.0037 versus 0.0003 in the global population from gnomAD V2.1.1 (fig. S4B and tables S1 to S3). Thus, ~0.001% of Africans would be expected to have a partial deficiency of pre-TCRα (~1/73,000 individuals). In various databases, the p.Asp51Ala variant is more frequent in individuals from South Asia and the Middle East, whose MAF is ~0.01. By contrast, the MAF for the global population from gnomAD V2.1.1 is about 0.002 (fig. S4C and tables S1 to S3). In these populations—which together account for almost two billion individuals—the p.Asp51Ala allele can be regarded as “common” (MAF >1%). Thus, 1/1000 to 1/10,000 Middle Eastern and South Asian individuals would be predicted to have a partial form of recessive pre-TCRα deficiency. We analyzed the exomes of two Iranian kindreds carrying the homozygous p.Asp51Ala variant and estimated that the most recent common ancestor carrying the variant lived about 8000 years ago (95% confidence interval: 2511-29,430 years). This finding suggests that there is no strong depletion of individuals homozygous for *PTCRA* in these populations. Thus, considering only the p.Asp51Ala and p.Tyr76Cys alleles, ~1/180,000 individuals worldwide may have a partial form of pre-TCRα deficiency. In particular, the p.Asp51Ala variant is found in the homozygous state in 1/1000 to 1/10,000 individuals in the populations of South Asia and the Middle East.

### Homozygosity for the Asp51Ala allele is a risk factor for autoimmunity

We investigated the impact of the p.Asp51Ala variant on immunological phenotypes by analyzing the reported phenotypes of individuals homozygous and heterozygous for this variants among the South Asians included in the UK Biobank. The frequencies of autoimmunity (~20%) and hypothyroidism (~10%) codes were similar in individuals heterozygous for p.Asp51Ala and controls (table S4). By contrast, three (75%) homozygous carriers had autoimmunity-related codes and one (25%) had a hypothyroidism-related code. Homozygote 1 suffered from hypothyroidism and lichen planus at the ages of 48 and 52 years, respectively. Homozygote 2 presented thrombocytopenia and Henoch–Schönlein purpura at the age of 50 years, and Homozygote 3 suffered from rheumatoid arthritis at the age of 50 years. No autoimmunity was reported in Homozygote 4, but he suffered from hypoxemic COVID-19 pneumonia at age 61. No lymphoproliferation was reported in any of the four homozygotes. We also analyzed the phenotype of the homozygotes identified in other cohorts (table S1). One of the two homozygotes in the Qatar Biobank was asymptomatic at the age of 46 years, and the other suffered from hypothyroidism with autoantibodies against thyroid peroxidase (TPO) at the age of 31 years. Two homozygotes were identified in a Saudi database and clinical data were available for only one, an otherwise healthy 38-year-old man with vitiligo. His 40-year-old sister was shown, by Sanger sequencing, to be homozygous for the variant, but was asymptomatic. In an Iranian database of individuals recruited on the basis of neurological phenotypes and Sanger sequencing data for the relatives of the proband, we identified three homozygous carriers of the p.Asp51Ala variant (P11, P12, and P13) (fig. S4D and Supplementary Text). None of these individuals presented unusual susceptibility to infection. However, two of the three children suffered from hypothyroidism. The thymic compartment of P11 (9 years old) contained tissue with abnormal properties on MRI, suggesting that the content of the thymus was abnormal (fig. S4E). Thus, evidence of autoimmunity was obtained for seven of the 11 (64%) homozygotes for whom clinical information was available. Finally, using the Centogene cohort, we identified 51 additional individuals homozygous for p.Asp51Ala (tables S5 and S6 and Supplementary Text). In this cohort, the association between autoimmunity and homozygosity for the p.Asp51Ala variant was confirmed, with an odds ratio (OR) of 5.02 relative to heterozygotes and WT subjects (95% CI=1.750054 to 11.816898, adjusted *P*=0.009965). Thus, homozygosity for the p.Asp51Ala variant appears to be a significant risk factor for the development of autoimmune disease in individuals of Middle Eastern and South Asian origin.

### Low CD3^+^ T cell counts in newborns with complete pre-TCRα deficiency

**Fig. 3.**
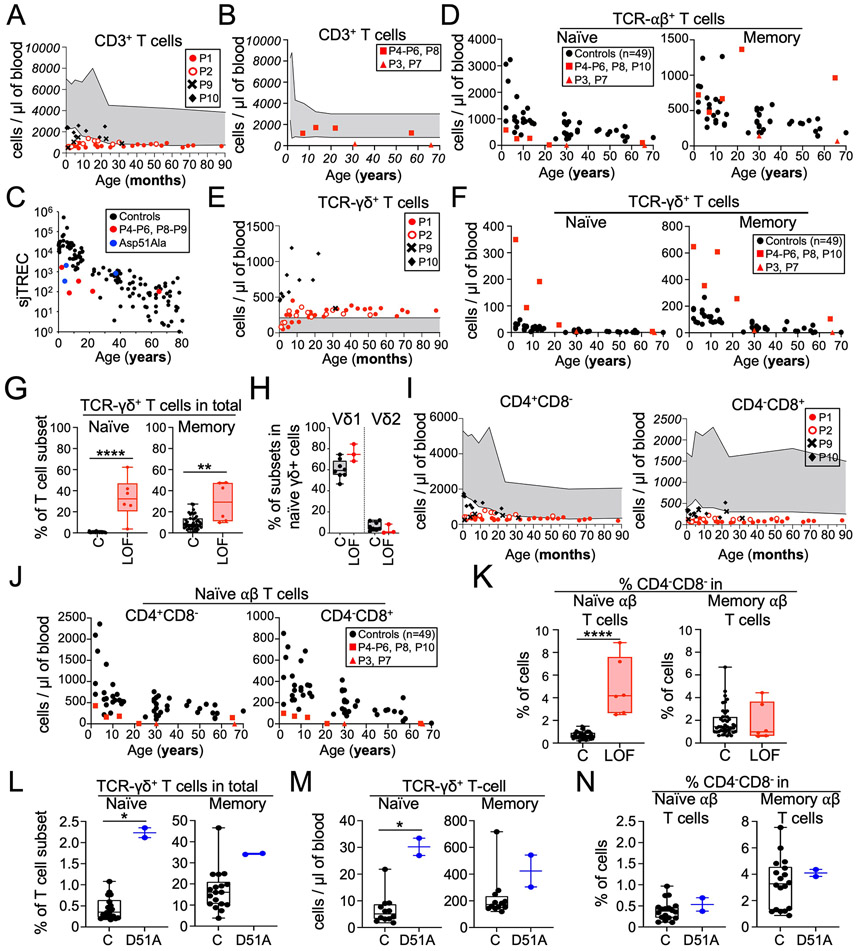
T cell immunophenotyping for patients with pre-TCRα deficiency (**A** and **B**) CD3^+^ T cell counts as a function of age. The control range is represented by the gray area. (**C**) Thymic function was assessed in pre-TCRα–deficient patients (red and blue dots) and healthy local controls (black dots; n=101). The concentration of sjTRECs in the blood (sjTREC/10^5^ PBMCs) is presented as a function of age. (**D**) TCRαβ^+^ T cell counts as a function of age for naïve and memory T cells. (**E**) TCRγδ^+^ T cell counts as a function of age. The control range is represented by the gray area. (**F**) TCRγδ^+^ T cell counts as a function of age for naïve and memory T cells. (**G**) Frequency of TCRγδ^+^ T cells among total naïve (CD3^+^CD45RA^+^CCR7^+^) and memory (defined as non-naïve CD3^+^) T cells from patients (P3-P6 and P8) and controls (n=46). (**H**) Frequency of TCRγδ1^+^ and TCRγδ2^+^ T cells among naïve TCRγδ^+^ T cells from patients (P4, P8, and P9) and controls (n=8). (**I**) Cell counts as a function of age, for CD4^+^CD8^−^ T cells and CD4^−^CD8^+^ T cells. The control range is represented by the gray area. (**J**) Naïve αβ T cell counts as a function of age, for CD4^+^CD8^−^ T cells and CD4^−^CD8^+^ T cells. αβ T cells are defined here as CD3^+^TCRγδ^−^ cells. (**K**) Frequency of CD4^−^CD8^−^ cells in αβ (defined here as CD3^+^TCRγδ^−^) naïve (right) and memory (left) T cells from patients (P3-P6 and P8) and controls. (**L** to **N**) Phenotyping of individuals homozygous for the p.Asp51Ala mutation (D51A) and controls (n=12-18). (L) Frequency of TCRγδ^+^ T cells among total naïve and memory T cells. (M) TCRγδ^+^ T cell counts for naïve and memory T cells. (N) Frequency of CD4^−^CD8^−^ cells among αβ (defined here as CD3^+^TCRγδ^−^) naïve (right) and memory (left) T cells. (B, D, F, and J) P3 suffered from severe enteropathy and was on rituximab treatment. P7 received chemotherapy for lymphoma. These two patients are therefore depicted with triangles. (G, H, and K to N) C: controls; LOF: patients homozygous for LOF variants; and D51A: individuals homozygous for the p.Asp51Ala mutation.

The asymptomatic pre-TCRα–deficient patients (P1, P2, P6, P8, and P9)—like the patient with a mild clinical presentation (P5)—had normal or near-normal distributions of leukocyte subsets other than T cells, and normal antibody responses to antigens (data S1; fig. S5; and Supplementary Text). By contrast, patients with clinical autoimmunity (P3, P4, and P7) were diagnosed with CVID and presented progressive cytopenia for multiple cell types. Pre-TCRα deficiency affects thymocyte differentiation in mice. We consequently investigated the blood T cell compartment of the patients. Except for P10, all patients (P1, P2, and P9) followed from birth displayed T cell lymphopenia early in life (Fig. 3A). Their total T cell counts remained stable over time, reaching counts at the low end of the normal range by the age of 3 years, when a physiological decline of CD3^+^ T cell counts is observed in normal individuals. Relative to age-matched controls, all patients other than P3 and P7 (aged 31 and 66 years respectively, both displaying progressive pancytopenia) had normal or near-normal blood counts of total CD3^+^ T cells at their most recent follow-up visit (Fig. 3, A and B). Moreover, all patients under the age of 30 years had low proportions of single-joint TRECs (sjTRECs) among PBMCs, suggesting poor thymic output (Fig. 3C). These data suggested that pre-TCRα deficiency impairs T cell development, resulting in low T cell counts in infancy, facilitating detection by newborn TREC level screening. However, the total T cell counts of the patients gradually increases, eventually reaching the normal range for age-matched controls (Fig. 3A). Moreover, these T cells proliferated normally upon mitogen stimulation in vitro (data S1)

### Patients with complete pre-TCRα deficiency have high naïve γδ and low naïve αβ T cell numbers

The mouse pre-TCRα is essential for αβ T cell development, but is dispensable for γδ T cell development (12). We therefore studied the impact of complete human pre-TCRα deficiency on the two major T cell lineages. Patients had lower blood counts of naïve αβ T cells than age-matched controls, but normal counts of memory αβ T cells (Fig. 3D). Total γδ T cell counts were high from early childhood (Fig. 3E). In children and adults, both naïve and memory γδ T cell counts remained normal to high (Fig. 3F). Accordingly, the proportion of γδ T cells among naïve T cells was higher in patients (median=32.3; range 3.7-62.3) than in controls (median=0.6; range 0.1-2.2) (Fig. 3G). However, the proportion of γδ T cells among memory T cells was above the upper limit of the control range only in P4, P5 and P10. The proportions of δ1^+^ and δ2^+^ γδ T cells among naïve T cells were normal in patients with pre-TCRα deficiency (Fig. 3H). Thus, pre-TCRα deficiency has different impacts on the thymic outputs of αβ and γδ T cells, impairing the production of αβ T cells and favoring the production of γδ T cells. Nevertheless, most circulating T cells (including naïve T cells) were TCRαβ^+^.

### Patients with complete pre-TCRα deficiency have normal memory αβ T cell counts and low MAIT cell counts

We then investigated the αβ T cell compartment in more depth. The infants had low total CD4^−^CD8^+^ and CD4^+^CD8^−^ T cell counts (Fig. 3I), which normalized between childhood and adulthood (fig. S6A). However, as expected from their low naïve αβ T cell counts, pre-TCRα–deficient children and adults had low counts of naïve CD4^+^ and CD8^+^ T cells (Fig. 3J). The low naïve T cell counts of the patients were accompanied by a higher proportion of both CD4 and CD8 effector memory T cells (fig. S6, B and C). The proportion of regulatory T cells (Tregs) among CD4^+^ T cells was in the range of controls for all patients (fig. S6D). Within the memory CD4^+^ T cell compartment, the frequencies of T helper subsets were within or near the control range (fig. S6E). Accordingly, comparisons with controls revealed no major differences in the production of T helper (Th)1 (IFN-γ), Th2 (IL-13), and Th17 (IL-17A) cytokines by the patients’ memory CD4^+^ T cells following stimulation (fig. S6F). In addition, pre-TCRα–deficient patients had lower levels of CD161^+^TCRVα7.2^+^ mucosal-associated invariant T (MAIT) cells than controls and normal frequencies of invariant natural killer T cells (iNKT) among T cells (fig. S6G). The frequency of TCRVα7.2^+^ cells was low among memory αβ T cells, but normal among naïve αβ T cells (fig. S6H), suggesting that the low frequency of MAIT cells was not due to impaired V(D)J rearrangement. Thus, patients with complete pre-TCRα deficiency have low total naïve αβ T cell counts, normal αβ T memory-cell counts from childhood onward, and a low frequency of MAIT cells.

### Patients with complete pre-TCRα deficiency have a high proportion of CD4^−^CD8^−^ DN αβ T cells among naïve T cells

Blood γδ T cells typically have a CD4^−^CD8^−/lo^ phenotype, defining a T cell lineage that does not pass through the CD4^+^CD8^+^ DP stage in the thymus. In TCR-transgenic mice expressing TCRαβ at the DN stage in the thymus, a small abnormal population of TCRαβ cells with a CD4^−^CD8^−^ phenotype is observed in the periphery (4, 30-32). Fate mapping has shown that these cells do not pass through the CD4^+^CD8^+^ DP stage. Instead, they are thought to use the γδ differentiation pathway despite their expression of a TCRαβ (33). We therefore investigated whether a fraction of TCRαβ^+^ cells in the periphery in pre-TCRα deficient patients harbored the same phenotype. We found no difference in the frequency of CD4^−^CD8^−^ cells among memory T cells from controls and patients (Fig. 3K). However, the frequency of CD4^−^CD8^−^ DN cells among naïve TCRαβ^+^ T cells from pre-TCRα–deficient patients was higher (median=4.2%; range: 2.5 to 8.9%) than that in age-matched controls (median=0.6%; range 0.2 to 1.5%) (Fig. 3K). These CD4^−^CD8^−^ DN cells did not have high levels of HLA-DR or CD38 expression and were, therefore, probably not chronically activated (fig. S6I) (34). Moreover, only small proportions of naïve CD4^−^CD8^−^TCRαβ^+^ T cells from the pre-TCRα–deficient patients expressed MAIT (CD161^+^TCRVα7.2^+^), iNKT (TCRVα24-Jα18^+^), Treg (CD127^−^CD25^+^), or intraepithelial lymphocyte (IEL) markers (CLA, CD103, NKG2C, and NKG2A), suggesting that most DN αβ T cells from the patients do not belong to an unconventional αβ T cell subset (fig. S6J). Thus, Pre-TCRα deficiency is associated with an approximately eightfold increase in the proportion of CD4^−^CD8^−^ T cells in the naïve TCRαβ^+^ T cell compartment. As in mice (4), DN αβ T cells in humans may therefore develop via an alternative T cell differentiation pathway.

### Low TREC levels and a high proportion of γδ T cells among the naïve T cells of p.Asp51Ala homozygotes

We tested the hypothesis that homozygosity for the hypomorphic p.Asp51Ala variant affects T cell differentiation. We determined the sjTREC levels of three patients (P11-P13). These levels were low in the two youngest patients (Fig. 3C). We also performed extensive immunophenotyping on these two children, which showed their counts and proportions of myeloid, B, and NK cells to be normal (fig. S7, A to D). The T cell counts of these patients were within the normal range for age-matched controls, as were the proportions of naïve and memory T cell subsets, and other T helper subsets, Tregs, iNKT, and MAIT cells (fig. S7, E to I). Nevertheless, the counts and proportions (among naïve T cells) of blood γδ T cells were higher in the p.Asp51Ala homozygotes than in controls (Fig. 3, L and M). By contrast to the findings for patients with complete pre-TCRα deficiency (Fig. 3K), the proportion of CD4^−^CD8^−^ cells in the naïve αβ T cell compartment was normal (Fig. 3N). Compared to patients with complete pre-TCRα deficiency, p.Asp51Ala homozygotes generally had a narrower but still distinctive immunological phenotype, with higher proportions of γδ T cells among naïve T cells. This was reminiscent of mice with mutations that affect similar charged residues of pre-TCRα (28, 29).

### Pre-TCRα deficiency impairs the generation of TCRαβ^+^ but not TCRγδ^+^ T cells in vitro

**Fig. 4.**
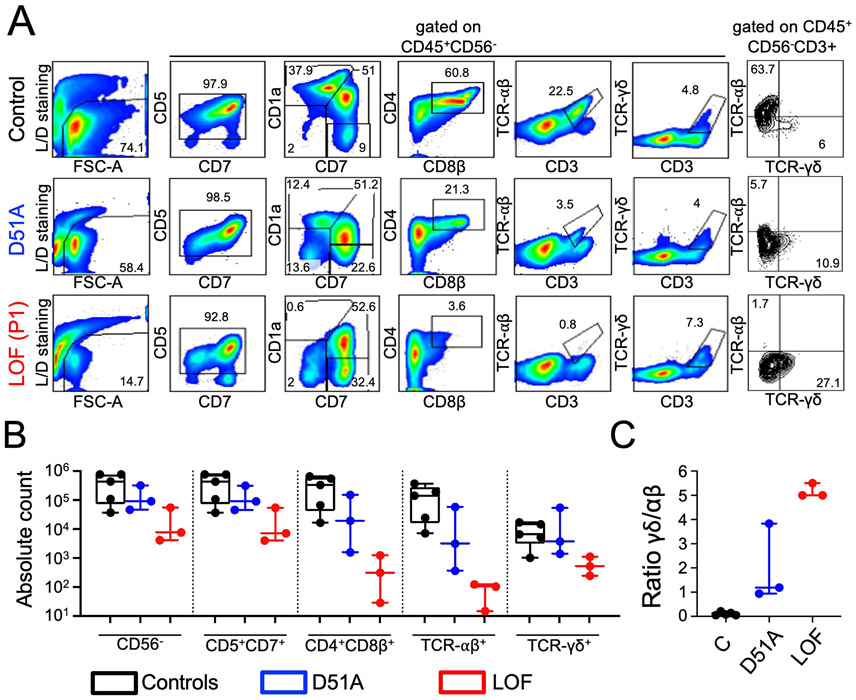
Impaired generation of TCRαβ^+^ T cells in pre-TCRα–deficient ATOs. In vitro T cell differentiation from positively selected peripheral blood CD34^+^ cells obtained from a healthy control, three patients with the p.Asp51Ala variant (D51A) and three patients with LOF *PTCRA* mutations (P1, P5, P6) after 5 weeks of culture in the ATO system. (**A**) Flow cytometry plots showing the expression of early and late T cell differentiation markers (CD7, CD5, CD1a, CD4, CD8β, TCRαβ, TCRγδ, and CD3) following gating on LIVE/DEAD^−^CD45^+^CD56^−^ cells. The data shown correspond to one control, one p.Asp51Ala patient and P1. (**B**) Plots of absolute counts/ATO for the various stages of T cell differentiation, for the cells isolated from the ATOs. (**C**) Bar graphs showing the ratio of absolute counts of TCRγδ^+^ cells to absolute counts of TCRαβ^+^ cells per ATO. C: controls, LOF: patients homozygous for LOF variants, D51A: individuals homozygous for the p.Asp51Ala variant.

We assessed the impact of the patients’ *PTCRA* genotype on the early stages of T cell differentiation by isolating blood CD34^+^ cells from three pre-TCRα–deficient patients and five healthy controls, and inducing their differentiation in vitro in an artificial thymic organoid (ATO) system (Fig. 4) (35). After 5 weeks of culture, control CD34^+^ cells remained highly viable (around 74%) (Fig. 4A). Efficient differentiation into CD4^+^CD8^+^ DP cells (mean ~52% of CD45^+^CD56^−^ cells), TCRαβ^+^CD3^+^ SP cells (~23%), and TCRγδ^+^CD3^+^ cells (~1.8%) was observed. By contrast, after 5 weeks of culture, viability was much lower for the CD34^+^ cells isolated from all three pre-TCRα–deficient patients (7 to 39%). The differentiation of these cells into T cells was impaired, with a block at the CD7^+^CD1a^+^CD4^−^CD8β^−^ DN stage and an almost total absence of CD4^+^CD8^+^ DP cells (mean ~2% of CD45^+^CD56^−^ cells) and TCRαβ^+^CD3^+^ SP cells (~0.5%). However, a significant fraction of the cells were TCRγδ^+^CD3^+^ (~3.5%). A determination of absolute counts per ATO of cells at various stages of differentiation confirmed the deficit of CD4^+^CD8β^+^ DP and TCRαβ^+^CD3^+^ cells and the presence of a significant number of TCRγδ^+^ cells in the three pre-TCRα–deficient patients studied, relative to controls (Fig. 4, A and B). In particular, the ratio of TCRγδ^+^ cells to TCRαβ^+^ cells was markedly higher in the pre-TCRα–deficient patients (~5) than in controls (~0.1) (Fig. 4C). Finally, ATOs generated with CD34^+^ cells from the three patients homozygous for the p.Asp51Ala variant had a phenotype intermediate between those of the controls and pre-TCRα–deficient patients (Fig. 4). Thus, complete pre-TCRα deficiency almost completely abolishes human αβ T cell differentiation in vitro, whereas partial deficiency due to p.Asp51Ala homozygosity has a milder impact.

### Sequencing of the *TRAD* locus in αβ T cells reveals an enrichment in proximal TCRδ1 and a depletion of distal MAIT cell rearrangements

**Fig. 5.**
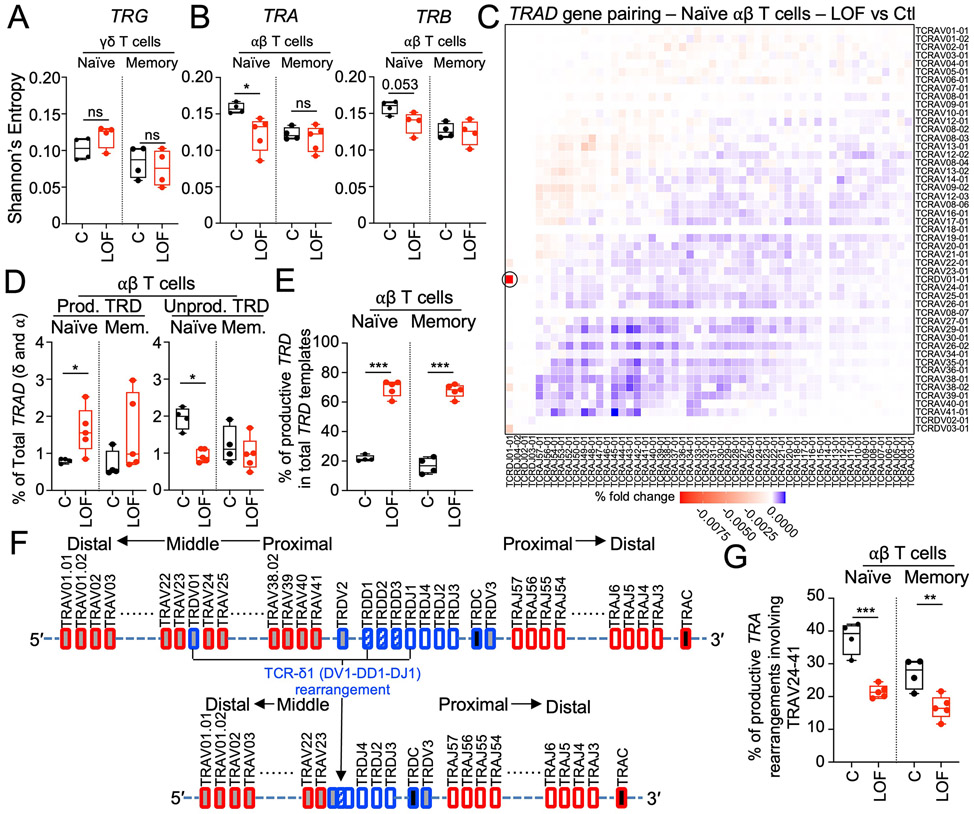
Biases in the *TRAD* rearrangement repertoire indicate that TCRα chains are mostly generated by rearrangement of a TCRδ1 template in pre-TCRα–deficient individuals. (**A**) Shannon’s entropy for TCRγ rearrangements in naïve and memory γδ T cells from controls (black; n=4) and pre-TCRα–deficient individuals (red; P1, P2, P4, and P8). (**B**) Shannon’s entropy for TCRα and TCRβ rearrangements in naïve and memory αβ T cells from controls (black; n=4) and pre-TCRα–deficient individuals (red; P1, P2, P4, P8, and P9). (**C**) Heatmap of paired gene rearrangements at the *TRAD* locus for naïve αβ T cells from four controls compared with five pre-TCRα–deficient individuals (P1, P2, P4, P8, and P9). The red color highlights V-J gene pairings overused in patients and the blue color highlights V-J gene pairings overused in controls. The TCRδ1 (*TRDV1*:*TRDJ1*) rearrangement is indicated with a black circle. (**D**) Fraction of TCRδ rearrangements in total productive *TRAD* rearrangements from sorted naïve and memory αβ T cells from controls (black; n=4) and pre-TCRα–deficient individuals (red; P1, P2, P4, P8, and P9). (**E**) Fraction of productive TCRδ rearrangements among total TCRδ rearrangements in naïve and memory αβ T cells from controls (black; n=4) and pre-TCRα–deficient individuals (red; P1, P2, P4, P8, and P9) (red). (**F**) Schematic representation of the *TRAD* locus before and after TCRδ1 rearrangement. (**G**) Percentage of productive TRA rearrangements involving *TRAV24-41* in sorted naïve and memory αβ T cells from controls (black; n=4) and pre-TCRα–deficient individuals (red; P1, P2, P4, P8, P9). Unpaired *t* tests were used for comparisons in panels A, B, D, and E.

The unexpectedly modest impact of pre-TCRα deficiency on αβ T cell development in vivo raised the question of how these cells developed in the absence of a major TCR component during the β-selection process. In patients with complete pre-TCRα deficiency, the circulating αβ and γδ TCR repertoire diversities were slightly low and normal, respectively, (Fig. 5, A and B; fig. S8A; table S7; and Supplementary Text). We then investigated whether the patients displayed preferential usage of productive V-J rearrangements at the *TRAD* locus in genomic DNA from purified naïve and memory αβ T cells. The most common productive V-J recombination at the *TRAD* locus in the naïve and memory αβ T cells of the patients was *TRDV01:TRDJ01* (i.e., TCRδ1) (Fig. 5C and fig. S8B). The percentages of productive and nonproductive *TRD* rearrangements among total *TRAD* rearrangements were significantly higher and lower, respectively, in the patients’ naïve αβ T cells than in those of the controls (Fig. 5D). Productive *TRD* rearrangements (involving any *TRDV*) consequently accounted for ~70% of the total *TRD* rearrangements detected in the αβ T cells of patients with complete pre-TCRα deficiency but only ~20% of those in healthy controls (*P*<0.0001) (Fig. 5E and fig. S8C). An analysis of the *TRAD* locus from purified naïve and memory αβ T cells showed a depletion of the *TRAV* genes removed during TCRδ1 rearrangement (*TRAV24*-*TRAV41*) in the productive TRAD rearrangements in the αβ T cells of patients relative to controls (Fig. 5, C, F and G, and fig. S8B). As a result, *TRAV* genes distal to *TRDV01* (*TRAV01*-*TRAV23*) were enriched in the patients’ αβ T cells (Fig. 5C and fig. S8B). TCRδ1 accounted for ~70% of total naïve γδ T cells (Fig. 3H), so such a pattern would be expected for TCRα repertoires preferentially rearranged from a TCRδ1 template, with TRAV23 becoming the most proximal *TRAV* gene following successful TCRδ1 (*TRDV01*:*TRDJ01*) rearrangement (fig. S8D). Thus, our *TRAD* repertoire analysis suggests that, in absence of pre-TCRα, TCRα rearrangements preferentially occur from a productive TCRδ1 template.

### TCRδ1 is not a surrogate for pre-TCRα

**Fig. 6.**
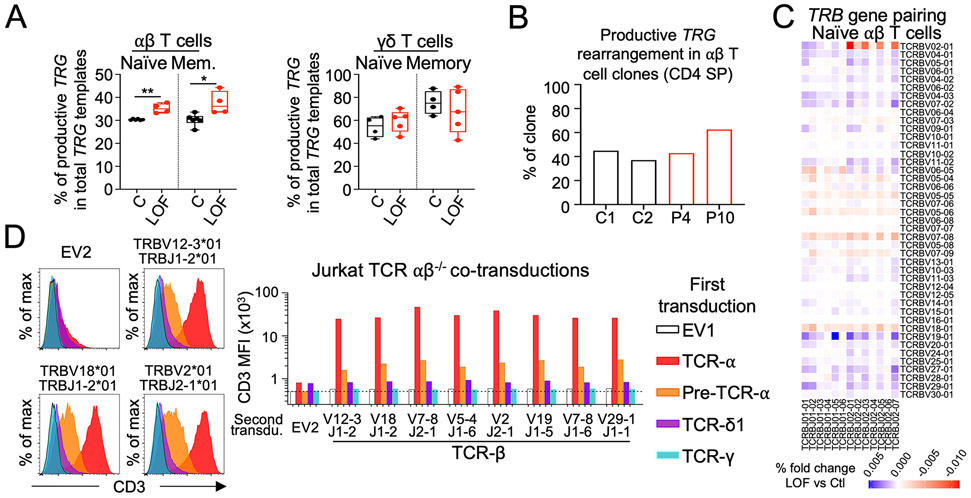
γδ^+^ thymocytes do not preferentially differentiate into αβ T cells in the absence of pre-TCRα, and TCRδ1 cannot act as a surrogate for pre-TCRα. (**A**) Fraction of productive TCRγ among total TCRγ templates in sorted naïve and memory αβ (left) or γδ (right) T cells from controls (black, n=4) and pre-TCRα–deficient individuals (red, P1, P2, P4, P9). Unpaired *t* tests were used for all comparisons. (**B**) Fraction of expanded αβ T cell clones from two controls and two pre-TCRα–deficient individuals with a productive TRG rearrangement at the gDNA level. Of note ~90% of these clones were CD4^+^CD8^−^. (**C**) Heatmap of paired gene rearrangements of the *TRB* locus for naïve αβ T cells from controls compared with five pre-TCRα–deficient individuals (P1, P2, P4, P8). The red color highlights V-J gene pairings overused in patients and the blue color highlights V-J gene pairings overused in controls. (**D**) TCRαβ–deficient Jurkat cells were stably transduced with an empty plasmid or with a plasmid encoding TCRα, pre-TCRα, TCRδ1 or TCRγ. Each of the resulting cell lines was then cotransduced with another empty plasmid or with a plasmid encoding one of eight selected TCRβ chains. The expression of CD3 at the cell surface was evaluated by flow cytometry. Representative flow cytometry histogram plot for three independent experiments. Representative flow cytometry data are shown on the left. A recapitulative bar graph of the MFI for CD3 for each cell line is shown on the right.

Having excluded the possibility that most αβ T cells preferentially differentiate from γδ^+^ thymocytes in the absence of pre-TCRα (Fig. 6,A and B; tables S8 and S9; and Supplementary Text), we hypothesized that TCRδ might act as a surrogate for pre-TCRα in the formation of a pre-TCR complex with specific TCRβ rearrangements and CD3. Consistent with this hypothesis, we found that the TCRβ repertoire was biased in patients with pre-TCRα deficiency, with an enrichment in rearrangements involving the middle *TRBV* genes and any *TRBJ* gene or involving the distal *TRBV02-1* gene and any *TRBJ02* gene (Fig. 6C and fig S8, E and F). We transduced TCRαβ–deficient Jurkat cells with TCRδ1, pre-TCRα, TCRα, or TCRγ cDNA. These stable cell lines were cotransduced with an empty vector or one of eight selected TCRβ chains, and CD3 stabilization at the cell surface was assessed by flow cytometry. TCRδ1 and TCRα alone stabilized low amounts of CD3 on the cell surface, whereas neither pre-TCRα, TCRβ, nor TCRγ alone could stabilize CD3 expression at detectable levels on the cell surface. Relative to transduction with single chains, we observed no enhancement of CD3 stabilization following cotransduction with TCRδ1 or TCRγ together with any of the tested TCRβ chains, including the TCRβ chain with the rearrangement frequently found in pre-TCRα–deficient patients and a TCRβ chain previously suggested to stabilize TCRδ1 expression (36). By contrast, pre-TCRα or TCRα stabilized CD3 expression at high levels on the cell surface following cotransduction with any TCRβ construct (Fig. 6D). Thus, in this system, TCRδ1 and TCRγ are unable to replace the pre-TCRα to stabilize TCRβ expression at the cell surface.

### Longitudinal study of peripheral T cells in 1-to-24-week-old *Ptcra*^−/−^ mice

**Fig. 7.**
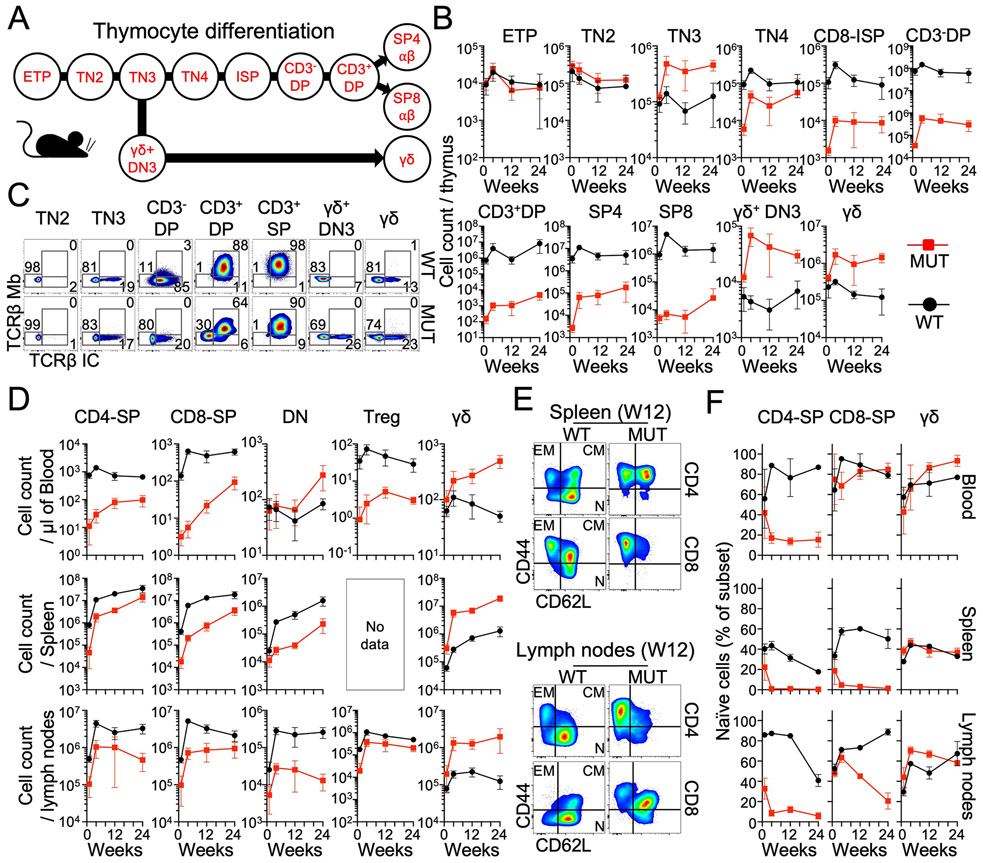
Longitudinal studies of *Ptcra*^−/−^ mice. (**A**) Schematic representation of thymocyte differentiation stages in mice. (**B**) Cell counts per thymus in *Ptcra*^−/−^ mice and WT mice aged 0-24 weeks, for the various thymocyte developmental stages. (**C**) Intracellular (IC) and membrane (Mb) expression of TCRβ for the indicated thymocyte subsets from *Ptcra*^−/−^ mice (bottom panel) and WT mice (upper panel). Cytometry data representative of 6 *Ptcra*^−/−^ mice and 6 WT mice are shown. (**D**) Counts of cells per microliter of blood or per spleen or per LN for *Ptcra*^−/−^ mice (red) and WT mice (black) aged 0-24 weeks for the indicated T cell subsets, including CD4-SP (CD3^+^TCRγδ^−^CD4^+^CD8^−^), CD8-SP (CD3^+^TCRγδ^−^CD4^−^CD8^+^), DN (CD3^+^TCR^−^γδ^−^CD4^−^CD8^−^), Treg (CD3^+^TCRγδ^−^CD4^+^CD8^−^CD25^+^), and γδ (CD3^+^TCRγδ^−^CD4^+^CD8^−^). (**E**) Representative flow cytometry plots of naïve and memory cell staining for CD4 and CD8 αβ T cells from the spleen and LNs of 12-week-old WT (black) and *Ptcra*^−/−^ mice (red). EM: effector memory; CM: central memory; N: Naïve. (**F**) Frequency of naïve cells among CD4-SP and CD8-SP αβ T cells, and of γδ T cells in the indicated tissue of *Ptcra*^−/−^ mice (red) and WT mice (black) from 0-24 weeks of age. (B, D and F) The data shown are the mean and standard deviation of 4 to 6 animals at each age.

We found that the peripheral αβ T cell counts of pre-TCRα–deficient humans normalized with age due to the physiological decrease in T cell counts with age in healthy individuals and an accumulation of memory αβ T cells in the patients. Four-week-old *Ptcra*^−/−^ mice have been reported to have 5% normal T cells in the LNs (12). However, T cell dynamics in the thymus and periphery have not been studied during aging. We therefore sought to reassess and extend mouse immunophenotyping longitudinally by studying the thymus, blood, spleen, and LNs of 1-, 4-, 12- and 24-week-old *Ptcra*^−/−^ and control mice. The skewed thymocyte differentiation observed in pre-TCRα–deficient mice remained stable in the aging thymus between the ages of 1 month and at least 6 months (Fig. 7, A to C, and Supplementary Text). Contrasting with the previous report of 5% normal T cells, we found that the CD4^+^ and CD8^+^ αβ T cell counts in the LNs of 4-week-old mice corresponded to 23% and 14% the normal level, respectively (Fig. 7D) (12). CD4^+^ αβ T cell counts were 39% and 26% the normal values in 12- and 24-week-old *Ptcra*^−/−^ mice, respectively, whereas CD8^+^ αβ T cell counts were 14% and 45% the normal values in 12- and 24-week-old *Ptcra*^−/−^ mice, respectively. Circulating CD4^+^ αβ T cell counts in *Ptcra*^−/−^ mice were 2%, 11%, and 15% the normal level, whereas circulating CD8^+^ αβ T cell counts were 1%, 5%, and 15% normal levels in 4-, 12- and 24-week-old mice, respectively (Fig. 7D). Splenic CD4^+^ αβ T cell counts in *Ptcra*^−/−^ mice were 17%, 18%, and 40% the normal values, whereas splenic CD8^+^ αβ T cell counts were 3%, 5%, and 19% the normal values in 4-, 12- and 24-week-old mice, respectively (Fig. 7D). Treg counts mirrored the counts of total CD4^+^ αβ T cells in the LNs and the blood (Fig. 7D). The increase in CD4^+^ and CD8^+^ αβ T cell counts in the periphery in aging *Ptcra*^−/−^ mice was driven by an accumulation of memory T cells, except for blood CD8^+^ T cells, which remained mostly naïve in 24-week-old mice (Fig. 7, E and F). The γδ T cell counts of *Ptcra*^−/−^ mice were higher than those of control mice at all timepoints with a greater increase in the spleen (10-tos20-fold between weeks 4 and 24 (W4 and W24)) and LNs (7-16-fold between W4 and W24) than in the blood (2-to-10-fold increase between W4 and W24) (Fig. 7D). As in humans, circulating αβ CD4^−^CD8^−^ DN T cells were more abundant in *Ptcra*^−/−^ compared with WT mice (Fig. 7D). Splenic and LN αβ CD4^−^CD8^−^ DN T cell counts did not significantly differ between *Ptcra*^−/−^ and mice, however. Thus, CD4^+^ and CD8^+^ αβ T cell counts increase in aging *Ptcra*^−/−^ mice mostly due to an expansion of the memory T cell compartment. These findings are consistent with our pre-TCRα–deficient patient data, highlighting the importance of studying aging animals when exploring thymic phenotypes.

## Discussion

The mouse and human *PTCRA* genes were discovered in 1994 and 1995, respectively, and the first pre-TCRα–deficient mice were described in 1995 (12, 37, 38). We report here 10 humans from seven kindreds and three distant ancestries with AR complete pre-TCRα deficiency. Given the severity of αβ T cell deficiency in pre-TCRα–deficient mice (12), it was expected that pre-TCRα–deficient humans would suffer from life-threatening infections in infancy. To our surprise, this was not the case, and six of our 10 patients, aged 2 to 65 years, have remained healthy. The remaining four patients have exhibited severe infection, lymphoproliferation, or autoimmunity beginning between the ages of 13 to 25 years. This relatively mild clinical phenotype is likely due to an age-dependent accumulation of normal numbers of diverse, functional memory αβ T cells. With hindsight, these findings do not conflict with the reported role of pre-TCRα in mice. Indeed, the impact of the *Ptcra*^−/−^ genotype on thymocytes and peripheral T cells have not been studied in aging mice and young mice, which survived in pathogen-free conditions, and have not been challenged with pathogens. As in humans, we observed a progressive increase in mouse blood αβ T cell counts with age, driven by memory cell accumulation. These findings highlight the need for caution when extrapolating phenotypes from mutant mice, which are often studied at a young age and in a narrow range of experimental conditions, to humans (39, 40). They also suggest that it can be productive to revisit mouse phenotypes based on human studies. In both mice and humans, αβ T cells can develop in the absence of pre-TCRα.

These findings raise questions about how diverse naïve αβ T cells develop in the absence of pre-TCRα. Our first hypothesis was that early productive proximal *TRA* rearrangements may permit αβ T cell development (41). However, we observed a depletion of productive *TRAD* rearrangements involving proximal *TRAV* genes (TRAV24-TRAV41) and an enrichment in rearrangements involving distal *TRAV* genes (TRAV1-TRAV23) in the patients’ naïve αβ T cells. Moreover, an abnormal enrichment in the productive TCRδ1 (*TRDV01:01*-*TRDJ01:01*) rearrangement was observed in αβ T cells from patients. As *TRAV* segments preferentially recombine with symmetric *TRAJ* segments (proximal V with proximal J, distal V with distal J) (42, 43), the TCRα repertoire observed in the absence of pre-TCRα—with a depletion of rearrangements involving proximal *TRAV* and an enrichment in rearrangements involving distal *TRAV*—suggests that these TCRα rearrangements occurred preferentially with a TCRδ1 template (fig. S8D). We therefore tested the hypothesis that TCRδ permits αβ T cell development. However, we found that TCRδ1 was unable to act as a surrogate for pre-TCRα in the formation of a pre-TCR. Moreover, similar to controls, most CD4^+^ SP αβ T cell clones from the patients did not carry a productive *TRG* rearrangement, suggesting that most of the patients’ T cells were unlikely to have differentiated directly from γδ^+^ thymocytes. These findings call for alternative hypotheses that may account for αβ T cell differentiation in the absence of pre-TCRα, which are consistent with the associated rearrangement bias observed at the *TRAD* locus.

We also identified two alleles affecting residues interacting with TCRβ responsible for partial pre-TCRα deficiency in homozygotes. Homozygosity for the p.Tyr76Cys variant affects about 1/76,000 individuals in sub-Saharan Africa. Homozygosity for p.Asp51Ala is more common, affecting between 1/1000 and 1/10,000 individuals in South Asian and Middle Eastern countries. The p.Asp51 residue was previously identified as potentially crucial for pre-TCRα function due to its conservation and negative charge (28). We show that this amino acid plays a crucial role in pre-TCRα dimerization with TCRβ. Humans homozygous for p.Asp51Ala have a partial form of pre-TCRα deficiency. They have normal cell counts of blood αβ T cell subsets, but high counts of naïve γδ T cells and low sjTREC levels. This phenotype is reminiscent of that of transgenic mice with substitutions in the extracellular domain of pre-TCRα, which have a phenotype intermediate between those of WT and *Ptcra*^−/−^ mice (29). The clinical phenotype of individuals with partial pre-TCRα deficiency is milder than that of individuals with complete pre-TCRα deficiency. The patients are asymptomatic or display isolated autoimmune manifestations. Homozygosity for hypomorphic mutations of *PTCRA* should be considered in patients with isolated autoimmunity, particularly the p.Asp51Ala substitution in individuals of South Asian or Middle Eastern origin. Collectively, our findings demonstrate that human pre-TCRα is largely redundant for αβ T cell development, but that its complete or partial deficiency can result in late-onset clinical manifestations (including autoimmunity in particular) with incomplete penetrance.

## Materials and Methods

### Informed consent

Written informed consent was obtained from legally authorized representatives in accordance with the Declaration of Helsinki. The study was approved by the ethics committee of INSERM (RCB 2010-A00634-35 et 2008-A01078-47), the UZ/KU Leuven ethical committee for research (reference number s58466), the Children’s Hospital of Philadelphia Institutional Review Board, and Tokyo Medical and Dental University (G2000-103). For studies of in vitro T cell differentiation and high-throughput sequencing of TCR repertoires, patients gave consent to participate in protocol 18-I-0128 approved by the NIH IRB and registered at www.clinicaltrials.gov as protocol NCT03610802.

### Whole-exome sequencing, whole-genome sequencing, and Sanger sequencing

P1, P9, and P10 underwent whole-exome sequencing as part of clinical care after a presumptive positive result for neonatal SCID screening. As a sibling of P1, P2 underwent neonatal SCID screening (presumptive positive result) and confirmatory testing for the familial mutations identified in P1. Commercial whole-genome sequencing (Macrogen) was performed for P3, P4, P5, and P7, due to their clinical history of immunodeficiency or autoimmunity. P6 and P8 were identified by regular Sanger sequencing for familial segregation analysis. The frequency of the p.Asp51Ala (Chr6(GRCh37):g.42890858A>C)) variant was evaluated in different cohorts including the South Asians from the UK biobank (44), 800 Qataris from the Qatar Genome Program (QGP) and Qatar Biobank (QBB) longitudinal study (45), Kuwaitis (46), Iranians (neurological phenotypes only), and Saudis (patients with suspected Mendelian diseases and their parents).

### Founder effect analysis for the p.Leu99Hisfs*68 and p.Asp51Ala variants

The occurrence of homozygosity for the p.Leu99Hisfs*68 and p.Asp51Ala variants in different kindreds suggested a founder effect. An analysis of the whole-exome sequencing data revealed that P4 (Kindred C) and P5 (Kindred D), both of whom are homozygous for p.Leu99Hisfs68*, have a homozygous haplotype around *PTCRA* and encompassing a 1.38 Mb region corresponding to 73 SNVs in common. The ESTIAGE method estimated the age of the most recent common ancestor (MRCA) of the two patients at 60 generations (95% CI (19-258 generations)) (47). Assuming a generation time of 27 years (48), the MRCA of these patients with the p.Leu99Hisfs68* mutation would have lived about 1600 (513-6966) years ago. Similarly, an analysis of the whole-exome sequencing data of P11 (Kindred H) and P13 (Kindred I), both homozygous for p.Asp51Ala, showed that they had a homozygous haplotype around *PTCRA* encompassing a 580 kb region corresponding to 39 SNPs in common. The ESTIAGE method estimated the age of the most recent common ancestor (MRCA) of these two patients at 301 generations (95% CI (93-1090 generations)) (47). Assuming a generation time of 27 years (48), the MRCA of these patients with the p.Asp51Ala mutation would have lived about 8000 (2511-29,430) years ago.

### Analysis of the UK Biobank data

We used the UK Biobank plink-formatted population-level exome original quality functional equivalent exome files for *n*=454,713 individuals (field 23155, with genotypes set to missing when read depth was <7 for single-nucleotide variations). For the UK Biobank cohort, the participants were aged 50 to 87 years as of 2021, and 55% were female. We further restricted our analysis to participants with South Asian ancestry, using field 21000. We considered a participant to be of South Asian ancestry if they had one of the following data codes for field 21000: 3001 (Indian), 3002 (Pakistani), 3 (Asian or Asian British), 3003 (Bangladeshi), or 3004 (any other Asian background). ICD10 codes and associated dates were collected from inpatient data (category 2000), cancer registries (category 100092) and first occurrences (category 1712), defined as the earliest occurrences of ICDs in the general practice, inpatient and death data, at three-digit resolution. The ICD10 codes used for autoimmune diseases were as follows: E03 (hypothyroidism), E10 (type 1 diabetes), M35 (Sjogren’s disease), G73.7 (Addison’s disease), K90.0 (celiac disease), M33 (dermatomyositis), M34 (systemic sclerosis), E05.0 (Graves’ disease), G35 (multiple sclerosis), M06 (rheumatoid arthritis), G70 (myasthenia gravis), D51.0 (pernicious anemia), M32 (systemic lupus erythematosus), D69.6 (autoimmune cytopenia), L80 (vitiligo), and L40 (psoriasis).

### Analysis of the Centogene Biodatabank

Exome and genome analyses performed at Centogene up to April 21, 2023 were analyzed (Centogene started exome and genome sequencing in 2014 and 2016, respectively). Only variants with a base coverage ≥10, read frequency ≥30, and variant quality ≥220 (only for exome sequencing) were retained for further analyses. We then selected all related genetic information for the variants NM_138296.3:c.152A>C (PTCRA p.(Asp51Ala)) and NM_138296.3:c.227A>G (PTCRA p.(Tyr76Cys)). We extracted information concerning the patient’s year of birth, sex, country of origin, family relationship, reported genetic test results of ES/GS-tested individuals, and HPO-encoded clinical information from our database when available. All the available de-identified data were aggregated at individual level.

We calculated p.(Asp51Ala) allele frequencies and their binomial confidence intervals by country and geographic region. For the phenotypic analysis, we stratified the cohort by PTCRA p.(Asp51Ala) genotype (homozygotes (HOM), heterozygotes (HET), and wild-type (WT)) and counted the number of occurrences per individual of any HPO term from a predefined list of 24 autoimmunity-related terms (table S5). We analyzed the difference in the proportions of individuals with a matching autoimmune-related phenotype (having at least one of 24 predefined HPO terms) by PTCRA p.(Asp51Ala) genotype. We tested the hypothesis of an association between the individual’s PTCRA p.(Asp51Ala) genotype and the presence of an autoimmune-related phenotype by enrichment analysis. Briefly, we calculated the odds ratio of having a positive match to the autoimmunity-related HPO terms from the predefined list, comparing all PTCRA p.(Asp51Ala) genotype groups in Fisher’s exact test. Statistical analyses and figures were produced with RStudio (version 2023.03.1 Build 446, Posit Software, rstudio.com), using R Statistical Software (version 4.3.0, R Core Team 2023, R-project.org) and the tidyverse package (version 2.0.0, Posit Software, tidyverse.org).

### RNA-seq analysis of sorted human thymocyte subsets

Data for biological triplicates of RNA-seq performed on sorted primary human thymic T cell subsets (Thy1/DN1, Thy2/DN2, Thy3/DN3, ISP4/ISP, DP early, DP late, and single-positive SP8 and SP4) were downloaded with the SRA toolkit and the fastq-dump v2.9.6 tool (dataset accession number BioProject: PRJNA741323) (16). The sequence reads were aligned with the human hg38 reference genome assembly with HISAT2 v2.2.1, using the -k 1 function (49). The principal pre-TCRα isoforms were detected with a combination of cufflinks v2.2.1 de novo transcript assembly (50) and the manual curation of transcript databases: Ensembl hg38 v96 and NCBI Refseq genes v110. Expression was estimated for five pre-TCRα isoforms, along with the full gene list in Ensembl v96, with kallisto v0.46.1, for each sample (51). Estimated normalized isoform expression, expressed in transcripts per million (TPM), was used to compare expression levels across thymocyte developmental stages and to calculate the abundance of isoform A relative to the other isoforms. The aligned reads were converted to BAM format with samtools v1.14 and the triplicates were combined with the *merge* function and loaded onto the Integrated Genome Viewer for figure preparation (52, 53).

### Sanger sequencing and TA cloning

Genomic DNA was obtained from whole blood from the patients. The *PTCRA* mutations identified by WES were checked by amplifying the corresponding gDNA regions with a recombinant *Taq* polymerase (Thermo Fisher Scientific). PCR products were purified by centrifugation through Sephadex G-50 Superfine resin (Merck) before and after the sequencing reaction, which was performed with the BigDye Terminator Cycle Sequencing Kit (Applied Biosystems) and the primers previously used to amplify the region of interest. Purified sequencing products were analyzed with an ABI Prism 3500 apparatus (Applied Biosystems) and aligned with the genomic sequence of *PTCRA* (Ensembl) with Serial Cloner 2.6 software. We checked that the compound mutations found in P7 and P8 really were in two different alleles by cloning the PCR amplicons from the gDNA of these patients with the TOPO TA cloning kit (Thermo Fisher Scientific) and using them for the one-shot transformation of TOP10 chemically competent *Escherichia coli* cells (Thermo Fisher Scientific). PCR with the M13 primers supplied with the TA cloning kit was performed on individual colonies before sequencing.

### Cell culture

HEK293T cells were cultured in DMEM (#61965059, Gibco) supplemented with 10% fetal bovine serum (Sigma-Aldrich). TCRα–deficient JR3.11 Jurkat cells and TCRαβ–deficient J76 Jurkat cells were cultured in RPMI (# 61870044, Gibco) supplemented with 10% fetal bovine serum (25). All cell lines were cultured at 37°C under an atmosphere containing 5% CO_2_. For transfection, HEK293T cells were plated at a density of 8×10^5^ cells per well in six-well plates. PBMCs were isolated from whole-blood samples by Ficoll-Hypaque centrifugation (Amer-sham-Pharmacia-Biotech).

### Plasmids and transient transfection

The C-terminally Myc/DDK-tagged pCMV6 empty vector and the human *PTCRA* expression vectors were purchased from Origene (NM_138296; #RC215794). Constructs carrying mutant alleles were generated by direct mutagenesis with the CloneAmp Hifi premix and polymerase (#639298, Takara). The resulting PCR products were digested with *Dpn*I (#R0176L, New England Biolabs) for 1 hour at 37°C, amplified in competent *E. coli* cells (#C3019H, New England Biolabs), and purified with a Maxiprep kit (#12663, Qiagen). Isoform B of pre-TCRα and the mutant allele lacking exons 1 to 3 were obtained by opening the Isoform A WT vector by PCR with primers flanking the region to be deleted and then using the Quick Blunting^™^ Kit (#E1201L, New England Biolabs) and the Quick Ligation^™^ Kit (#M2200S, New England Biolabs) as recommended by the manufacturer. Lentiviral plasmids carrying the various *PTCRA* variants were generated by inserting the cDNA from the pCMV6 plasmids into an empty pTrip-SFFV-ΔNGFR vector (modified pTRIP-SFFV-mtagBFP-2A; addgene, plasmid #102585). This was achieved by digesting the empty pTrip-SFFV-ΔNGFR vector with *Xho*I and *Bam*HI for 1 hour at 37°C. The cDNA of interest was amplified by PCR and inserted into the vector by homologous recombination with the In-Fusion^®^ HD Cloning Kit according to the manufacturer’s instructions (#638911, Takara). Lentiviral plasmids pTrip-SFFV-ΔNGFR encoding TCRα, TCRδ, TCRγ, or TCRγδ and pTrip-SFFV-GFP encoding various forms of TCRβ were synthesized by TwistBioscience, after onboarding our empty vector plasmid. HEK293T cells were transiently transfected in the presence of the X-tremeGENE 9 DNA Transfection Reagent **(#**6365787001, Roche), in accordance with the manufacturer’s instructions.

### Choice of TCRα, TCRβ, TCRγ and TCRδ1 cDNA sequences

The TCRα sequence was obtained from addgene plasmid #128544 (after removal of the intron). The choice of TCRβ was based on a comparison of the TRB repertoire of naïve αβ cells from patients and controls. The TRBV2*01∣TRBJ2-1*01, TRBV7-8*01∣TRBJ2-1*01, TRBV5-4*01∣TRBJ1-6*01 and TRBV7-8*01∣TRBJ1-6*01 rearrangements were selected because they were found to be overused in patients’ cells. Conversely, the TRBV19*01∣TRBJ1-5*01 and TRBV29-1*01∣TRBJ1-1*01 rearrangements were selected because they were less frequently detected in the patients’ cells than in control cells. The TRBV12-3*01∣TRBJ1-2*01 and TRBV18*01∣TRBJ1-2*01 TCRβ chains were selected because they are the chains expressed in the Jurkat and DN-D41 cell lines, respectively (54). These two cell lines were used as controls. It has been suggested that DN-D41 expresses the TCRδ1/TCRβ heterodimer at the cell surface (36). The full-length TCRδ1, TCRγ and TCRβ sequences were assembled with stitchR software (55). The TCRγ sequence used was the example data provided by stitchR. The CDR3 of the TCRδ1 and TCRβ sequences were randomly picked from the most frequent αβ or γδ T cell clones of the patients with the V-J of interest according to our TCR bulk sequencing data. The full sequences of all the TCRs used in this study are provided in the supplementary materials.

### Artificial gene and exon trapping for the c.58G>C and c.58+5G>A alleles

We cloned *PTCRA* gDNA from a control, as described in Fig. 2A. The inserted *PTCRA* gDNA sequence, extending from the 5′ UTR to the end of exon 2, is represented in blue (exons) and gray (intron 1). Briefly, *PTCRA* gDNA containing exon 1 and the first 909 nucleotides of intron 1 was amplified with CloneAmp Hifi premix (Takara), the forward primer 5′- GAGATCTGCCGCCGCGTAGAAGGCAGTCTTGTGGGTGC-3′, and the reverse primer 5′-AAGGAACTCAGTTCCTCCAGGACTCAACCTCCAGA-3′. Similarly, *PTCRA* gDNA containing the last 724 nucleotides of intron 1 and exon 2 was amplified with the forward primer 5′-GGAACTGAGTTCCTTGAGAGCAGGGACAATGACTTAC-3′ and the reverse primer 5′-CTCGAGCGGCCGCGTACGCGTTGACAGATGCATGGGCTGTGTAC-3′. The Infusion Cloning kit (Clontech) was used to insert both PCR products between the ASIS1 and *Mlu*1 cloning sites of the pCMV6 entry vector (Origene) by homologous recombination. The c.58+G>C or c.58+5G>A mutation was generated by mutagenesis. We extracted mRNA from HEK293T cells after 24 hours of transfection with the WT, c.58G>C or c.58+5G>A exon-trapping vectors. The cDNA for the *PTCRA* transcript was amplified with a recombinant *Taq* polymerase (Thermo Fisher Scientific), a forward primer 5′-TAGAAGGCAGTCTTGTGGGTGC-3′ binding to the 5′ UTR of *PTCRA*, and a reverse primer 5′-CATTTGCTGCCAGATCCTCTT-3′ binding to the in-frame C-terminal Myc/DDK tag. The PCR products were then cloned with the TOPO TA cloning kit (Thermo Fisher Scientific) and used for the one-shot transformation of TOP10 chemically competent *E. coli* cells (Thermo Fisher Scientific). Splice variants of *PTCRA* from individual colonies were amplified with the M13 primers supplied with the TA cloning kit before sequencing and alignment with the *PTCRA* cDNA (NM_138296.2), with SnapGene software used to identify alternative splicing variants. We screened 82 colonies for the WT *PTCRA* exon-trapping vector, 65 for the c.58G>C *PTCRA* exon-trapping vector, and 83 for the c.58+5G>A *PTCRA* exon-trapping vector.

### mRNA purification, and RT-qPCR

Total RNA was extracted from the indicated cells with the RNeasy Extraction Kit (Qiagen). RNA was reverse-transcribed with the SuperScript II reverse transcriptase (Thermo Fisher Scientific) and oligo-dT primers (Thermo Fisher Scientific). We then performed qPCR with the Applied Biosystems Assays-on-Demand probes/primers specific for PTCRA-FAM (Hs00300125_m1) on 100 ng cDNA. The data were normalized relative to the expression (ΔCt) of GUS (13-glucuronidase-VIC, 4326320E) and are expressed as 2^−ΔCt^ values.

### Cell lysis and immunoblotting

The exon-trapping primers were designed to retain the translation frame of the Myc/DDK tag from the pCMV6 plasmid (Fig. 2A). The end of exon 2 was, therefore, fused to a Myc/DDK tag for detection of the artificial fusion protein. Total protein was extracted from HEK293T cells after 24 hours of transfection with the WT, c.58G>C or c.58+5G>A exon-trapping vectors. We assessed the expression of *PTCRA* variants by extracting total protein from HEK293T cells 48 hours after transfection with the various pCMV6 plasmids encoding the PTCRA variants. Total protein extracts were obtained by incubating cells with lysis buffer (50 mM Tris, pH 7.4, 150 mM NaCl, 2 mM EDTA, and 0.5% Triton X-100). A mixture of protease and phosphatase inhibitors was added to the buffers: aprotinin (10 μg/ml; Sigma-Aldrich), PMSF (1 mM; Sigma-Aldrich), leupeptin (10 μg/ml; Sigma-Aldrich), protease inhibitor cocktail (Sigma-Aldrich). After 30 min of lysis at 4°C, the cells were centrifuged for 10 min at 16,000*g*, and the supernatant was collected for immunoblotting. For each variant, we separated 20 μg of total protein by SDS-PAGE and immunoblotting was performed with antibodies against the DDK Tag (1:3000, HRP-coupled, M2, #A8592, Sigma-Aldrich), PTCRA (1:3000, PA5-95578, Invitrogen), GAPDH (1:5000, FL335, #sc47724 HRP, Santa Cruz Biotechnology) or vinculin (1:5000, EPR8185, #ab129002, Abcam). Staining was detected with the Clarity Western ECL substrate (Biorad, #1705061) or SuperSignal West Femto (ThermoScientific, #34096) with ChemiDoc MP (Biorad).

### Lentivirus production and transduction

The lentiviruses used for the transduction of TCRα–deficient JR3.11 Jurkat cells and TCRαβ–deficient J76 Jurkat cells were produced by transfecting HEK293T cells with pCMV-VSV-G (0.2 μg) (56), pHXB2 env (0.2 μg; NIH-AIDS Reagent Program; #1069), psPAX2 (1 μg; gift from D. Trono; Addgene plasmid #12260) and a vector containing the sequence for transduction. The vectors containing the sequences for transduction were pTrip-SFFV-ΔNGFR (empty vector), pTrip-SFFV-GFP (empty vector), pTrip-SFFV-ΔNGFR-PTCRA-WT, the other pTrip-SFFV-ΔNGFR vectors containing the *PTCRA* variants studied, pTrip-SFFV-ΔNGFR-TCRα, pTrip-SFFV-ΔNGFR-TCRδ, pTrip-SFFV-ΔNGFR-TCRγ, pTrip-SFFV-ΔNGFR-TCRγδ and the pTrip-SFFV-GFP-TCRβ vectors. HEK293T cells were transfected in six-well plates and the medium was replaced after 6 hours of incubation. The virus-containing supernatant was collected and passed through a 0.2-μm filter 24 hours after the medium was changed. Protamine sulfate (8 μg/ml) was added to the virus-containing supernatant, which was then added to Jurkat cells (immediately after seeding), which were spinoculated for 2 hours at 1200*g* and 25°C. The cells were then cultured for 48 hours at 37°C under an atmosphere containing 5% CO_2_, without shaking. Transduction efficiency was then checked by flow cytometry with the GFP tag or an anti-CD271 antibody (#557196, BD, 1:500). Transduced cells were sorted with a magnetic MACS^®^ Column and the CD271 MicroBead Kit (#130-099-023, Miltenyi Biotec), as recommended by the manufacturer.

### Flow cytometry analysis of JR3.11 Jurkat cells and J76 Jurkat cells

Transduced JR3.11 Jurkat cells and J76 Jurkat cells were stained with antibodies against CD271 (#557196, BD, 1:500), CD3 (#555333, BD, 2:50), C1β TCR (#565776, BD, 2:50), or CD69 (#310912, Biolegend, 1:400) and incubated with the Aqua Live/Dead Cell Stain Kit (Thermo Fisher Scientific) for 1 hour at room temperature before analysis with a Gallios (Beckman Coulter) flow cytometer. All data were analyzed with FlowJo 10.5.3 software.

### Intracellular cytokine staining

Intracellular Cytokine Staining (ICS) was performed as previously described (57). Briefly, PBMCs were thawed in cRPMI (RPMI 1640 1X supplemented with 10% heat-inactivated fetal bovine serum (FBS), 1% L-glutamine and 1% Pen-Strep) and centrifuged before being resuspended at a concentration of 2 × 106 cells/ml in cRPMI. Cell pellets were resuspended in surface antibody cocktail (table S10) and incubated for 20 min. Cells were then permeabilized with permeabilization reagent (Invitrogen) and incubated for 20 min followed by a wash with PERM Buffer. Cell suspensions were centrifuged, and the pellets stained with intracellular antibody cocktail (table S10) for 60 minutes. Lastly, cells were washed with permeabilization buffer before being resuspended in 1.6% PFA to fix. The fixed cells were stored overnight at 4°C and analyzed on Aurora Spectral flow cytometer (Cytek).

### Mass cytometry (CyTOF)

CyTOF was performed with various strategies. One involved the use of whole blood in the Maxpar Direct Immune Profiling Assay, 30 Markers (Standard Biotools, ref: 201334), according to the manufacturer’s instructions. All the samples for whole-blood staining were processed within 24 hours of collection. P10, P12, and P13 were phenotyped by the same protocol but with a customized antibody panel (table S11). We investigated the T cell subsets, including IEL markers, with another CyTOF staining panel for cryopreserved samples (IEL panel, table S12). PBMCs were thawed and 4×10^6^ cells were immediately stained according to the Standard Biotools protocol. The antibodies against TCR Vδ1 and TCR Vδ2 were added after 10 min of staining with the other antibodies to prevent interference with the binding of the TCRγδ antibody. For both whole blood and IEL panels, cells were frozen at −80°C after iridium staining and stored at the same temperature until acquisition on a Helios machine (Standard Biotools). In addition to whole-blood immunophenotyping, we also performed immunophenotyping on cryopreserved PBMCs for some patients (table S13). Single-cell suspensions were centrifuged to obtain a cell pellet, which was then incubated with 20 μM lanthanum-139 (Trace Sciences)–loaded maleimido-mono-amine-DOTA (Macrocyclics) in PBS for 10 min at room temperature for live–dead discrimination (LD). Cells were washed in staining buffer and resuspended in surface antibody cocktail, incubated for 30 min at room temperature, washed twice in staining buffer, fixed, permeabilized with the FoxP3 staining buffer set (eBioscience), and subjected to intracellular staining for 60 min at room temperature. Cells were washed twice and then fixed by overnight incubation in 1.6% PFA (Electron Microscopy Sciences) solution supplemented with 125 nM iridium at 4°C. Before data acquisition on a CyTOF Helios flow cytometer (Standard Biotools), cells were washed twice in PBS and once in dH_2_O. Custom conjugation to isotope-loaded polymers was performed with the MAXPAR kit (Standard Biotools). The data were analyzed with OMIQ software.

### Luciferase reporter assays for autoantibodies against IFNs

The blocking activity of anti-IFN-α, anti-IFN-β, and anti-IFN-ω autoantibodies was assessed in a luciferase reporter assay, as described elsewhere (58).

### Protein array for assessing autoantibodies

Protein arrays (HuProt^™^ v4.0 from CDI laboratories) for assessing autoantibodies were performed as previously described (24).

### Single-cell RNA sequencing (5′ transcriptomics, αβ and γδ TCR)

CD3^+^ T cells were sorted by flow cytometry from the PBMCs of P1, P2 (two independent samples analyzed, collected at the ages of 12 and 18 months), P3, P4, and healthy controls matched for age. Cryopreserved peripheral blood mononuclear cells (PBMC) in R10 medium (RPMI 1640, 10% FBS, 2 mM L-glutamine, 100 U/ml of penicillin, and 100 μg/ml of streptomycin) were thawed and immediately centrifuged to obtain a cell pellet. The cells were then incubated for 15 min at 37°C under an atmosphere containing 5% CO_2_ in the presence of Benzonase (Millipore Sigma, cat. 70664) diluted 1:1000 in R10 medium. The cells were then washed once in R10 and once in FACS buffer (2% FBS in PBS). For staining, cells were resuspended in 50 μl of a mixture of LIVE/DEAD Fixable Blue Dead Cell Stain (cat. L34962) diluted 1:200 in PBS and anti-CCR7 APC-Cy7 antibody (Biolegend, cat. 353212) and incubated for 10 min at 37°C under an atmosphere containing 5% CO_2_. Cells were then labeled with 1 μl of oligonucleotide-linked hashing antibody (Totalseq-C, Biolegend) and stained by incubation with 50 μl of antibody cocktail diluted in Brilliant Stain Buffer (BD Biosciences, cat. 566349) for 20 min at room temperature. The antibody cocktail contained the following antibodies: anti-CD14 BV510 (Biolegend, cat. 301842), anti-CD19 BV510 (Biolegend, cat. 302242), anti-CD56 BV510 (Biolegend, cat. 362534), anti-CD8 BV785 (Biolegend, cat. 301046), anti-CD4 PECy7 (Biolegend, cat. 300512), anti-CD95 AlexaFluor 700 (Biolegend, cat. 305648), anti-PD-1 BV750 (Biolegend, cat. 329966), anti-CD69 FITC (Biolegend, cat. 310904), anti-CD40L BV421 (Biolegend, cat. 310824), anti-CD3 BUV805 (BD Biosciences, cat. 741999), anti-CD45RA PECF594 (BD Biosciences, cat. 562298), anti-CD25 BUV661 (BD Biosciences, cat. 741685), anti-CXCR3 BV711 (BD Biosciences, cat. 563156), anti-HLA-DR PECy5.5 (Invitrogen, cat. MHLDR18), anti-CXCR5 APC (Invitrogen, cat. 17-9185-42). The cells were washed twice with FACS buffer and resuspended in R10 for sorting. We sorted 12,000 T cells (CD3^+^CD14^−^CD19^−^CD56^−^Live/Dead^−^) from each sample with a BD FACSymphony S6 Cell Sorter instrument (BD Biosciences) running BD FACSDiva Software version 9.5.1 (BD Biosciences). Sorted cells were pooled four by four, and each pool was loaded in a different lane of the 10x Genomics Chromium Chip for sequencing. For the sequencing of single-cell V(D)J repertoires for sorted T cells, the cell suspension was loaded on the 10x Genomics Chromium Instrument according to the manufacturer’s protocol for the Next GEM Single-Cell 5′ Kit v1.1 (10x Genomics PN-1000165) to generate gel bead-in-emulsions and for GEM-RT and the amplification of total cDNA. Following purification with SPRIselect beads (Beckman Coulter), specific TCR targets were amplified from the cDNA with the PTCR1 primer (5′-AATGATACGGCGACCACCGAGATCTACACTCTTTCCCTACACGACGCTC-3′) and constant region primers: TRB (5′-TGCTTCTGATGGCTCAAACACAGCGACCT-3′), TRA (5′-TCTCAGCTGGTACACGGCAGGGTCAGGGT-3′), TRG (5′-GAAGGAAGAAAAATAGTGGGCTTGGGGGAAAC-3′), or TRD (5′-CACCAGACAAGCGACATTTGTTCC-3′) with a barcode and the P7 sequence added to the constant region primers. The Illumina-ready libraries were sequenced by paired-end MiSeq with 2×300 base-pair reads to obtain VDJ sequences.

### Evaluation of TCR entropy and TCR chain combinations in scRNA-seq data

We processed 10x single-cell transcriptome libraries with Cellranger (v6.1.1) and Seurat (v4.0.4). The TCRαβ and TCRγδ libraries were demultiplexed and cell barcodes were assigned with Minnn (v10.1). TCR libraries were annotated with MiXCR (v3.0.13) and then separated by subject. The numbers of αβ or γδ TCRs for the patients and controls were calculated by counting the numbers of cells expressing both *TRA* and *TRB* V-J genes and both *TRG* and *TRD* V-J genes. The final counts corresponded to the intersection of cells expressing combinations of *TRA*, *TRB*, *TRG* or *TRD* genes, accounting for 10,365, 21,755, 2233, and 1440 cells, respectively, for the controls (*n*=11) and 5963, 11,416, 2095, and 529 cells, respectively, for the patients (*n*=5). The diversity of α, β, γ, and δ TCRs was estimated by calculating Shannon’s entropy (H) index. Entropy was calculated by summing the frequencies of each clone (CDR3 amino-acid sequence) and multiplying by the base 2 logarithm of the same frequency over all cells expressing *TRA*, *TRB*, *TRG* or *TRD* V-J genes. Higher H-index values indicate a more diverse distribution of CDR3 clones (59).

### VirScan - phage immunoprecipitation-sequencing (PhIP-Seq)

Antibody profiling by phage immunoprecipitation-sequencing (PhIP-Seq) was performed on plasma samples from patients and controls as previously described (60).

### In vitro T cell differentiation in the artificial thymic organoid (ATO) system

In vitro T cell differentiation was studied by coculturing peripheral blood CD34^+^ cells with a DLL4-expressing stromal cell line (MS5-hDLL4) in the ATO system, as previously described (61), but with minor modifications. Briefly, CD34^+^ peripheral blood cells from five normal donors, three patients with partial pre-TCRα deficiency (P11-P13), and three patients with complete pre-TCRα deficiency (P1, P5 and P6) were positively selected with the CD34 MicroBead UltraPure kit (Miltenyi Biotec) on an AutoMACS Pro Separator. We mixed 1000-1500 CD34^+^ cells with 150,000 MS5-hDLL4 cells per ATO. Each ATO (5 μl) was then plated in a 0.4 μM Millicell Transwell insert and placed in one well of a six-well plate in 1 ml complete RB27 medium supplemented with rhIL-7 (5 ng/ml), rhFlt3-L (5 ng/ml), and 30 μM L-ascorbic acid 2-phosphate sesquimagnesium salt hydrate. Each insert contained a maximum of two ATOs. For the first 3 weeks of culture, the medium was also supplemented with 10 ng/ml of rhSCF. After 5 weeks in culture, MACS buffer (PBS supplemented with 0.5% BSA and 2 mM EDTA) was added to each well and ATOs were dissociated by manual pipetting. The cells were then collected into a pellet by centrifugation, resuspended in FACS buffer (2% FBS in PBS), counted, and stained with the antibody cocktail described in table S14. Events were acquired on a BD LSR II Fortessa flow cytometer (BD Biosciences, San Jose, CA) and analyzed with FlowJo software version 10.6.1 (FlowJo, LLC, Ashland, OR).

### Mouse experiments

Mice were bred under specific pathogen-free conditions in CIPHE animal facilities (agreement number: B1301407) and handled in accordance with institutional committee and European guidelines for animal care. C57BL/6 mice were purchased from Janvier Laboratories. Ptcratm1(icre)Hjf KO mice have been described elsewhere (62) and were rederived from the INFRAFRONTIER/EMMA archive (EM:08347). Multiparametric immunophenotyping was performed at the CIPHE-PHENOMIN (INSERM, US012) flow cytometry facility. Peripheral blood (PB) was collected by submandibular puncture into Microvette^®^ 500 K3 EDTA tubes (Sarstedt). Hematological analysis was performed on a Procyte Dx (IDDEX) machine, in accordance with the manufacturer’s recommendations. Peripheral blood leukocytes were analyzed with a Lyse No Wash protocol and 1X FACS Lysing Solution (BD Biosciences). Leukocytes from the spleen and thymus were extracted according to the protocol of the International Mouse Phenotyping Consortium (IMPC_IMM_002). Red blood cells were not lysed for thymic leukocyte preparations. Lymph node (LN) T cells were isolated from pooled inguinal, brachial, axillary, and submandibular LNs. Briefly, organs were disrupted with the OctoGentleMACS system (Miltenyi Biotec), using 600 Mandl units of collagenase D (Roche Life Science) and 30 μg of DNAse I (Sigma), for 20 min at room temperature. The cell suspension was filtered and the cells were counted. Red blood cells were lysed by incubation for 1 min at room temperature with ammonium-chloride-potassium (ACK) lysis solution (eBioscience). Before staining, the cells were incubated for 10 min on ice with an anti-CD16/32 (2.4G2) antibody to block Fc receptors. In all experiments, DAPI (Invitrogen) staining was used to exclude dead cells from the analysis. Multiparameter FACS acquisition was performed on a Fortessa LSRII SORP or Canto 10C system (BD Biosciences). The analysis was performed with FACSDiva 9.01 (BD Biosciences) software. Doublets were systematically excluded on the basis of side scatter (SSC) and forward scatter (FSC) parameters. The antibodies used for immunophenotyping are listed in table S15. The thymocyte subsets were defined as ETP (CD4^−^CD8a^−^CD3e^−^CD44^+^CD25^−^ckit^+^), TN2 (CD4^−^CD8a^−^CD3e^−^CD44^+^CD25^+^ckit^+^), TN3 (CD4^−^CD8a^−^CD3e^−^CD44^−^CD25^+^γδ^−^), TN4 (CD4^−^CD8a^−^CD3e^−^CD44^−^CD25^−^γδ^−^), ISP (CD4^−^CD8a^+^CD3e^−^CD44^−^CD25^−^γδ^−^), iDP (CD4^+^CD8a^+^CD3e^−^CD44^−^CD25^−^γδ^−^), mDP (CD4^+^CD8a^+^CD3e^+^CD44^−^CD25^−^γδ^−^), SP4 (CD4^+^CD8a^−^CD3e^+^CD44^−^γδ^−^), SP8 (CD4^−^CD8a^+^CD3e^+^CD44^−^γδ^−^), γδ TN3 (CD3e^+^CD25^+^γδ^+^) or γδ (CD3e^+^CD25^−^γδ^+^).

### High-throughput sequencing (HTS) of the human TCR repertoire from the gDNA of sorted naïve and memory T cells

PBMCs were stained with antibodies against CD3 (#565491, BD, 1:50), CD45RA (#130-092-247, Miltenyi Biotec, 2:50), CCR7 (#130-120-600, Miltenyi Biotec, 2:50), TCRγδ (#331218, Biolegend, 1:50), or TCRαβ (#555548, BD, 2:50) and incubated with the Aqua Live/Dead Cell Stain Kit (Thermo Fisher Scientific) for 30 min at room temperature. Naïve and memory αβ and γδ T cells were sorted with a FACSAria cell sorter (Becton Dickinson, San Jose, CA) on the basis of CD45RA and CCR7 expression. DNA extraction was performed with the DNeasy Blood & Tissue Kit (#69504; Qiagen). The rearranged *TRAD*, *TRB*, and *TRG* genomic loci were sequenced by Adaptive Biotechnologies (Seattle, WA) as a commercial service. The data were then analyzed with ImmunoSeq online tools (Adaptive Biotechnologies) and custom R scripts. The frequencies of productive and nonproductive *TRD, TRG, TRB*, and *TRA* rearrangements were analyzed for both unique and total *TRD, TRG*, *TRB*, or *TRA* sequences obtained from the sorted αβ and γδ T cell subsets. The frequency distributions for individual clonotypes (including *TRBV*-to-*TRBJ* pairing and *TRAV*-to-*TRAJ* pairing) were analyzed within unique sequences. Diversity indices were calculated and heat-map representations of the frequencies of individual *TRAV/TRDV* to *TRAJ/TRD* gene pairs and *TRBV*-to-*TRBJ* gene pairs were produced with R software version 4.2.0 (2022-04-22 ucrt) and the R packages Tidyverse (1.3.2) and Immunarch (0.6.9).

### High-throughput sequencing (HTS) of the human TRG locus from the gDNA of clonally expanded T cells

T cell clones were obtained by sorting naïve T cells (CD3^+^CD45RA^+^CCR7^+^TCRαβ^+^TCRγδ^−^) with a BD FACSAria III SORP cell sorter (Becton Dickinson, San Jose, CA) and DIVA 9.1 software. Cells were sorted, one cell per well, in 96-well plates containing 50 μl of ImmunoCult^™^-XF T Cell Expansion Medium (StemCell Technologies, REF #10981) supplemented with IL-2 (1 ng/ml) and ImmunoCult^™^ Human CD3/CD28/CD2 T cell Activator (StemCell Technologies ,REF #10990,1:40) per well. Every 2 days, fresh medium with IL-2 (1 ng/ml) was added to the cells. Clones were visible under a microscope 1 week after sorting. Clones were reactivated every three weeks with ImmunoCult^™^ Human CD3/CD28/CD2 T cell Activator (StemCell Technologies, #10990,1:80). DNA was extracted from clones with the DNeasy Blood & Tissue Kit (#69504 ; Qiagen). The *TRG* gene repertoire was investigated by next-generation sequencing (NGS). For library preparation, PCR was performed on 100 ng of genomic DNA with a published protocol (63), but with adaptation of the primers for a NGS version of the assay (table S16). Dual barcoding of the primers made the simultaneous multiplexing of samples possible. After library purification, sequencing was performed on an Illumina^™^ MiSeq platform. Sequencing data analysis, including demultiplexing, quality control and clonotype assignment, was performed with the Vidjil pipeline (https://www.vidjil.org). IMGT V-QUEST (https://www.imgt.org/IMGT_vquest/analysis) was used for further TRGV and TRGJ annotation and for CDR3 characterization.

### TREC levels

Single-joint T cell receptor excision circles (sjTRECs) were quantified by nested quantitative polymerase chain reaction (qPCR), with the primers and standard curve plasmid described by Dion et al. (64). The qPCR protocol was adapted as described in Roux et al. (65) using approximately 500 ng of purified gDNA for each quantification.

### Statistics

Analyses were performed with GraphPad Prism V10.1.1 software. Two-tailed Mann–Whitney tests or unpaired *t* tests were used for single comparisons of independent groups. In the corresponding figures, n.s. indicates not significant; ****P<0.0001, ***P<0.001; **P<0.01; and *P<0.05.

## Supplementary Material

Data_Table

Supplementary_Data
